# Studying the Geroprotective Properties of YAP/TAZ Signaling Inhibitors on *Drosophila melanogaster* Model

**DOI:** 10.3390/ijms24066006

**Published:** 2023-03-22

**Authors:** Denis A. Golubev, Nadezhda V. Zemskaya, Anastasia A. Gorbunova, Daria V. Kukuman, Alexey Moskalev, Mikhail V. Shaposhnikov

**Affiliations:** Laboratory of Geroprotective and Radioprotective Technologies, Institute of Biology, Komi Science Center, Ural Branch, Russian Academy of Sciences, 167982 Syktyvkar, Russia

**Keywords:** YAP/TAZ, *Drosophila*, aging, geroprotector

## Abstract

The transcriptional coactivators Yes-associated protein (YAP) and transcriptional coactivator with PDZ-binding motif (TAZ) are the main downstream effectors of the evolutionarily conserved Hippo signaling pathway. YAP/TAZ are implicated in the transcriptional regulation of target genes that are involved in a wide range of key biological processes affecting tissue homeostasis and play dual roles in the aging process, depending on the cellular and tissue context. The aim of the present study was to investigate whether pharmacological inhibitors of Yap/Taz increase the lifespan of *Drosophila melanogaster*. Real-time qRT-PCR was performed to measure the changes in the expression of *Yki* (*Yorkie*, the *Drosophila* homolog of YAP/TAZ) target genes. We have revealed a lifespan-increasing effect of YAP/TAZ inhibitors that was mostly associated with decreased expression levels of the *wg* and *E2f1* genes. However, further analysis is required to understand the link between the YAP/TAZ pathway and aging.

## 1. Introduction

With the increase in the average age of the population, preventing premature aging and treating age-related diseases has become a major concern in modern healthcare [1,2,3]. The promising approach to achieve this goal is to influence the major molecular mechanisms associated with aging which is the main risk factor for age-related diseases, in order to suppress pathological processes and activation of the defense systems of the cell and the body as a whole [4,5,6].

Recent studies demonstrate that damage to long-lived macromolecules, including extracellular matrix (ECM) proteins, make a significant contribution to the aging process [7,8]. The accumulation of non-enzymatic modifications by glycation, oxidation, and crosslinking of collagen and elastin, the major components of the ECM, occurs during aging [7,9]. The modifications of macromolecules affect the structural and physical properties of the ECM that increase the stiffness of tissues and reduce their viscoelasticity [10,11,12,13]. High ECM stiffness promotes activation of Yes-associated protein (YAP) and transcriptional coactivator with PDZ-binding motif (TAZ), the Hippo pathway effectors [14] that play a key role in the regulation of tissue homeostasis [15]. YAP/TAZ are transcription coactivators without DNA-binding activity that activate or repress target gene expression through interaction with various transcription factors [16]. ECM stiffness has been shown to regulate YAP/TAZ-driven gene transcription independent of the Hippo pathway [17] and make a significant contribution to the deregulation of tissue homeostasis during aging [18,19]. Due to YAP/TAZ regulating target genes involved in a wide range of key biological processes, such as modulation of nuclear integrity and functions [20], stem cell differentiation [21], cell proliferation [22], regeneration [23], innate immune response [24,25], and tumorigenesis [26], YAP/TAZ activity plays dual roles in the aging process depending on cellular and tissue context [19,20]. For example, reduced expression of connective tissue growth factor (CTGF), an established YAP/TAZ target gene, which is involved in tissue remodeling, has been reported to mediate collagen loss in chronologically-aged human skin [27]; whereas, persistent activation of CTGF can result in increased deposition of collagen and fibrotic conditions [28].

Despite a large amount of experimental data having demonstrated a decrease in YAP/TAZ activity during physiological aging [19,23,24,29], a number of studies have shown that several compounds with proven anti-aging properties such as resveratrol [30], rapamycin [31], metformin [32], and simvastatin [33] inhibit transcriptional activity of YAP/TAZ [19,34]. However, the effects of geroprotectors on YAP/TAZ activity in normal tissues have not yet been studied.

*Drosophila melanogaster* is one of the most studied and genetically tractable model organisms for investigating the mechanisms of aging and anti-aging interventions [35,36] using evolutionarily conserved aging-related signaling pathways as potential drug targets [37,38]. *Drosophila* Yorkie (Yki) is a homolog of mammalian YAP/TAZ [39,40] that allows the use of fly models in studies of the geroprotective properties of Yap/Taz inhibitors.

The aim of this study was to investigate whether pharmacological inhibitors of Yki/Yap/Taz could improve the survival of *D. melanogaster*. In this study, we investigated the effects of pharmacological inhibitors of Yki/Yap/Taz on *D. melanogaster* survival. The following substances, with previously established inhibitory effects on Yap/Taz activity, were used: Verteporfin (VP) [41,42,43], ML-7 hydrochloride (ML7) [44], Cytochalasin D (CD) [45,46,47], and AICAR (AI) [48].

## 2. Results

### 2.1. Expression Levels of YAP/TAZ Target Genes

Real-time qRT-PCR analyses of YAP/TAZ target genes expression provide direct quantitative estimation of YAP/TAZ transcriptional activity [49,50]. The analyzed targets of Yki included genes that promote cellular growth (*myc*) [51], genes involved in cell cycle progression (*CycE* and *E2F1*) [52,53], inhibitors of apoptosis (*Diap1*) [54], genes encoding ligands for different signaling pathways (*wg* and *vein*) [55,56], and modulators of signaling pathway activity (*dally*) [57].

**Figure 1:**
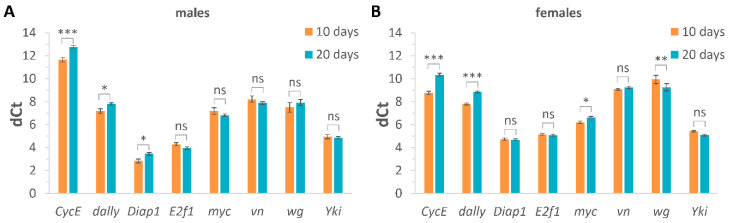
Age-related changes in the expression level of Yki target genes. Estimation of the expression level in males (**A**) and females (**B**) at the age of 10 and 20 days. Two-way ANOVA (age × gene) followed by post hoc Duncan test was used to compare differences in expression levels of genes between 10-day-old and 20-day-old flies, * *p* < 0.05, ** *p* < 0.01, *** *p* < 0.001, ns—not significant. The Ct (cycle thresholds) values are inversely proportional to the mRNA transcript levels. The delta Ct (dCt) values were calculated as the differences in Ct values for target genes and reference genes (*β-Tubulin* and *RpL32*). Higher dCt values represent a lower expression level. The error bars show standard errors. The experiments were performed in three biological replicates and three technical replicates (*n* = 3, repeated three times). The samples included 20 males and 10 females.

To study whether the expression levels of Yki target genes changed with aging, qRT-PCR was performed for male and female flies at the ages of 10 and 20 days (Figure 1, Table S1). Two-way ANOVA analysis (gene × age) showed a significant effect of the gene (*p* < 0.001), a significant effect of the age (*p* < 0.05), and a significant interaction (*p* < 0.001) in male and female flies (Figure 1, Table S2). Given that cycle threshold (Ct) values are inversely proportional to mRNA transcript levels, higher differences in Ct values (dCt) between target genes and reference genes represent a lower expression level. A Duncan post hoc test of the 10-day-old flies versus 20-day-old flies within each gene revealed a significant (*p* < 0.05) age-related decrease in the expression levels of *CycE*, *dally*, *Diap1* in males and *CycE*, *dally*, *myc* in females, but an age-related increase in the expression level of *wg* in females (*p* < 0.01). As most of the statistically significant changes indicated a decrease in the expression level (increase in dCt values) of the analyzed genes, this suggests a possible age-related decrease in the level of Yki transcriptional activity.

These results are consistent with previously published data which demonstrated an age-related decrease in the transcriptional activity of YAP/TAZ [19,23]. However, it should be noted that because of the complex crosstalk between YAP/TAZ and other signaling pathways, such as Wnt [58] and Notch [59], several of the used genes are not exclusive targets of the Yki pathway. Therefore, for a more convincing conclusion regarding age-related changes in Yki pathway activity, a more direct and specific read-out of Yki activity should be used in further research.

To assess whether treatment with various concentrations of substances was associated with the inhibition of YAP/TAZ transcriptional activity, the expression levels of Yki target genes were compared between treated and control groups of different ages within each inhibitor.

**Figure 2:**
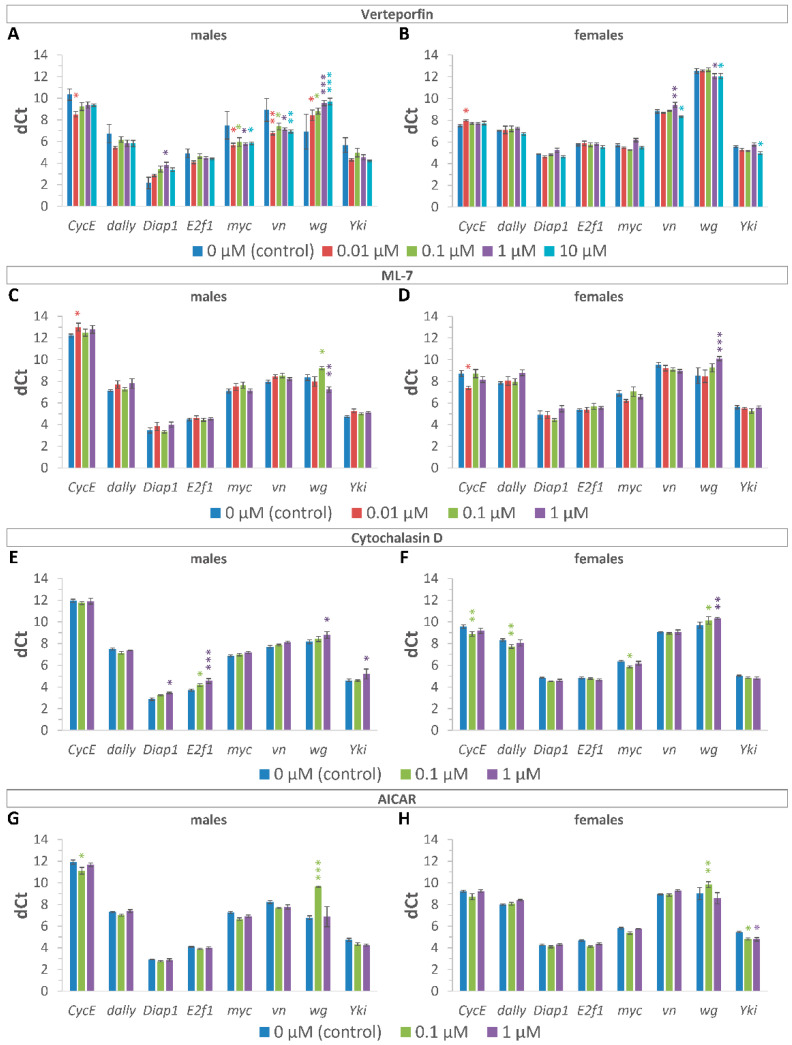
Effects of YAP/TAZ inhibitors on the expression level of Yki target genes. Estimation of the expression level in 10 day-old males and females after treatment with VP (**A**,**B**); ML7 (**C**,**D**); CD (**E**,**F**); AI (**G**,**H**). Two-way ANOVA (age × gene) followed by post hoc Duncan test was used to compare differences in expression levels of genes between 10-day-old and 20-day-old flies, * *p* < 0.05, ** *p* < 0.01, *** *p* < 0.001. The Ct (cycle thresholds) values are inversely proportional to the mRNA transcript levels. The delta Ct (dCt) values were calculated as the differences in Ct values for target genes and reference genes (*β-Tubulin* and *RpL32*). Higher dCt values represent a lower expression level. The error bars show standard errors. The experiments were performed in three biological replicates and three technical replicates (*n* = 3, repeated three times). The samples included 20 males and 10 females.

Two-way ANOVA (gene × concentration) showed that there were significant differences in expression levels among the different genes (*p* < 0.001, source of variation: gene) in 10-day-old male and female flies. The ANOVA also revealed significant differences in gene expression between the control animals and animals treated with inhibitors at different concentrations (*p* < 0.05, source of variation: concentration), except males and females treated with AI. Additionally, the ANOVA demonstrated that effects of the inhibitor treatment depended on the gene (*p* < 0.001, source of variation: interaction), except males treated with CD (Figure 2, Table S3).

Duncan test of the inhibitor-treated 10-day-old male flies versus control age- and sex-matched flies within each substance revealed a significant (*p* < 0.05) decrease in the expression level of *CycE* (0.01 μM ML7), *Diap1* (1 μM VP and 1 μM CD), *E2f1* (0.1–1 μM CD), *wg* (0.01–10 μM VP; 0.1 μM ML7; 1 μM CD), *Yki* (1 μM CD), but an increase (*p* < 0.05) in the expression of *CycE* (0.01 μM VP; 0.1 μM AI), *myc* (0.01–10 μM VP), *vn* (0.01–10 μM VP), *wg* (1 μM ML7), and *Yki* (0.01 and 10 μM VP) (Figure 2, Table S4). At the same time, the post hoc pairwise comparison of gene expression level in treated and control 10-day-old females demonstrated a decrease (*p* < 0.05) in the expression of *CycE* (0.01 μM VP), *vn* (1 μM VP), *wg* (1 μM ML7; 0.1–1 μM CD; 0.1 μM ML7), but an increase (*p* < 0.05) in the expression of *CycE* (0.01 μM ML7; 0.1 μM CD), *myc* (0.1 μM CD), *vn* (10 μM VP), *wg* (1–10 μM VP), and *Yki* (10 μM VP; 0.1–1 μM AI) (Figure 2, Table S5). All inhibitors have shown the ability to suppress the expression of at least one gene. The most characteristic effect of used inhibitors is the suppression of *wg* gene expression (except for females treated with VP).

The observed differences in the expression levels between different genes in individuals of the same sex and between the same genes in males and females are consistent with the transcriptional data from the FlyAtlas2 database [60], and reflect sex differences in gene expression of most of the studied genes, including Yki target genes.

To determine whether 20 days of treatment is more effective than 10 days of treatment for inhibition of YAP/TAZ transcriptional activity, the expression level of Yki target genes was compared between treated and control groups at the age of 20 days.

Two-way ANOVA (gene × concentration) showed that there was a significant effect of the gene (*p* < 0.001), a significant effect of the concentration (*p* < 0.05, except males treated with VP, ML7, AI), and a significant interaction (*p* < 0.05, except males treated with AI and females treated with ML7, CD, AI) in 20-day-old flies (Figure S1, Table S6).

Duncan post-test of the 20-day-old flies versus ones within each gene revealed a significant (*p* < 0.05) decrease in expression level of the *CycE* gene in female flies treated with VP (1 μM) and with CD (0.01 μM), but increased in males treated with ML7 (1 μM). The Duncan’s test also demonstrated a decrease (*p* < 0.05) in expression of the *wg* gene in males and females treated with VP (0.01 μM and 1 μM, respectively) and with ML7 (0.01 μM), as well as in females treated with CD (0.1 μM). However, *wg* expression increased in males treated with CD (0.1 μM) and females treated with VP (10 μM) and AI (0.1 μM) (Figure S1, Tables S7 and S8).

Thus, among four Yap/Taz inhibitors, the most pronounced effects on gene expression levels were detected in flies at the age of 10 days. Based on the obtained results, we used the concentration which showed the most significant effect on gene expression to determine the effect of substances on *Drosophila melanogaster* lifespan.

### 2.2. Effects on Survival

To address whether the used solvents have potential toxic effects on flies, we compared the lifespan between the control group treated with 100% (*v*/*v*) water and control groups that were treated with 100% (*v*/*v*) ethanol, 50% (*v*/*v*) solution of ethanol in water, and 10% (*v*/*v*) solution of Cyrene in water. Pairwise comparisons of the survival curves, median, and maximum lifespan (using Fleming–Harrington test and Fisher’s exact test with Bonferroni correction, respectively) revealed a positive effect of 100% (*v*/*v*) ethanol (in females), negative effect of 50% (*v*/*v*) ethanol solution (in males), and positive effect of 10% (*v*/*v*) Cyrene solution (in males and females) (Figure S2; Table S9). These results indicate that there are no acute toxic effects of the used solvents. A further analysis of the effects of Yap/Taz inhibitors was then conducted using different appropriate control groups.

According to the results of qRT-PCR analysis, the following concentrations of the YAP/TAZ inhibitors were chosen for the survival analysis: 0.01 μM and 0.1 μM—for VP, 0.1 μM and 1 μM—for ML7, CD, and AI. Two independent replicate experiments were completed for each group (Figures S3 and S4, Table S10).

**Figure 3:**
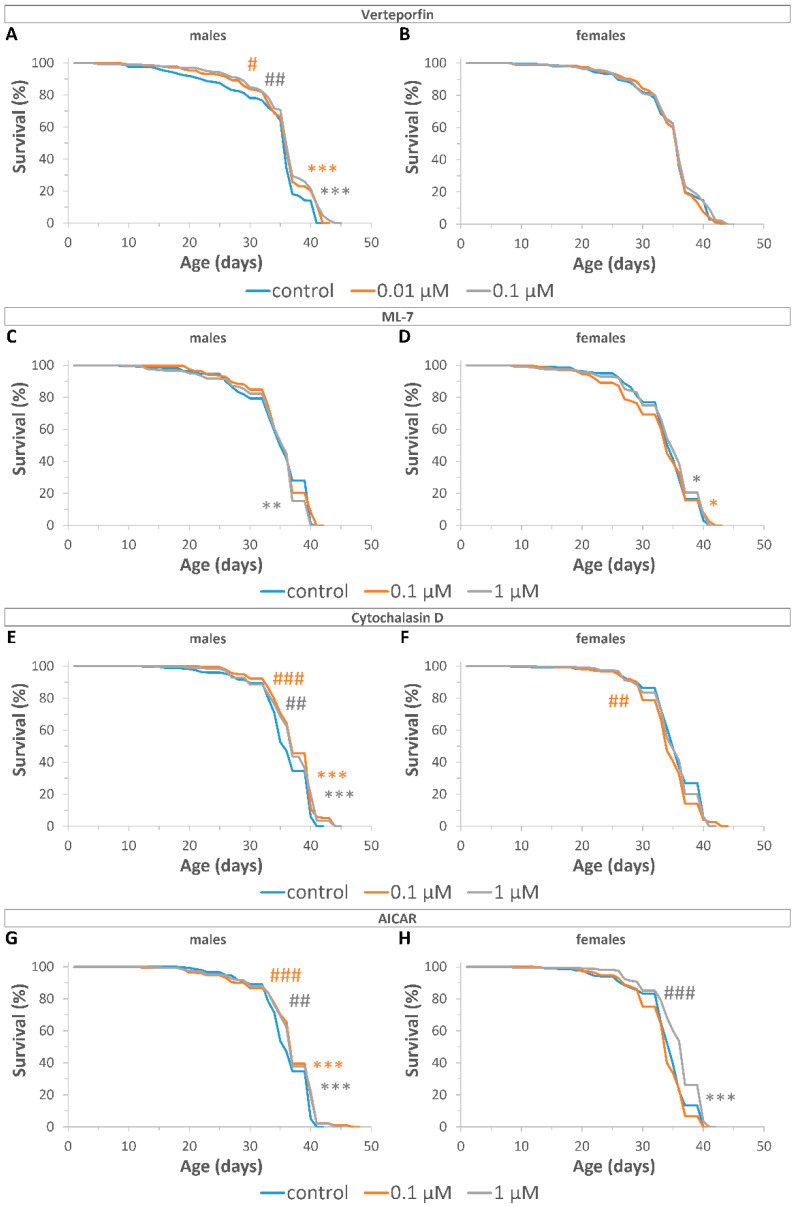
Effects of YAP/TAZ inhibitors on the lifespan of male (**A**,**C**,**E**,**G**) and female (**B**,**D**,**F**,**H**) flies after treatment with VP (**A**,**B**); ML7 (**C**,**D**); CD (**E**,**F**); AI (**G**,**H**). Fleming–Harrington test sensitive against early (^#^ *p* < 0.05, ^##^ *p* < 0.01, ^###^ *p* < 0.001) and later (* *p* < 0.05, ** *p* < 0.01, *** *p* < 0.001) differences were used to compare survival curves between inhibitor-treated and control flies. Bonferroni correction was used for multiple comparisons. Two independent experiments were performed: *n* = 287 (VP control, males); *n* = 306 (0.01 µM VP, males); *n* = 293 (0.1 µM VP, males); *n* = 290 (ML control, males); *n* = 277 (0.1 µM ML, males); *n* = 299 (1 µM ML, males); *n* = 290 (CD control, males); *n* = 324 (0.1 µM CD, males); *n* = 303 (1 µM CD, males); *n* = 293 (AI control, males); *n* = 321 (0.1 µM AI, males); *n* = 293 (1 µM AI, males); *n* = 282 (VP control, females); *n* = 305 (0.01 µM VP, females); *n* = 281 (0.1 µM VP, females); *n* = 300 (ML control, females); *n* = 274 (0.1 µM ML, females); *n* = 283 (1 µM ML, females); *n* = 292 (CD control, females); *n* = 293 (0.1 µM CD, females); *n* = 330 (1 µM CD, females); *n* = 323 (AI control, females); *n* = 310 (0.1 µM AI, females); *n* = 330 (1 µM AI, females).

To reduce incidental effects, we pooled the results of two replicates (Figure 3, Table S11). Using Fisher’s Exact test to compare median and maximum lifespan and Fleming–Harrington test to estimate early and later differences between survival curves of treated and control flies, we revealed the lifespan effects of YAP/TAZ inhibitors.

VP at concentrations of 0.01 μM and 0.1 μM increased the maximum lifespan (by 2%, *p* < 0.001), as well as reduced the rate of early (*p* < 0.05) and late mortality (*p* < 0.001) in males, but had no effect (*p* > 0.05) on the survival of females (Figure 3A,B, Table S11). ML7 at concentration of 1 μM increased late mortality (*p* < 0.01) in males, but at concentrations of 0.1 μM and 1 μM reduced late mortality (*p* < 0.05) in females (Figure 3C,D, Table S11). CD at concentrations of 0.1 μM and 1 μM increased median (by 3%, *p* < 0.05) and maximum (by3%, *p* < 0.01) lifespan in males, respectively. However, CD at a concentration of 0.1 μM increased the rate of early mortality (*p* < 0.01) in females (Figure 3E,F, Table S11). AI at concentration of 0.1 μM increased maximum lifespan (by 3%, *p* < 0.05), and at concentrations of 0.1 μM and 1 μM reduced the rate of early and late mortality (*p* < 0.001) in males. AI at concentrations of 0.1 μM and 1 μM increased median lifespan in females by 3%, *p* < 0.01 and by 6%, *p* < 0.001, respectively, and at a concentration of 1 μM reduced the rate of early (*p* < 0.001) and late mortality (*p* < 0.001) in females (Figure 3G,H, Table S11).

Thus, *Drosophila* treatment with YAP/TAZ inhibitors decreased late mortality, which indicates a geroprotective potential of this intervention.

### 2.3. Effects on Inflammatory Markers of Aging

Previously, it has been demonstrated that aging in *Drosophila* is accompanied by a considerable increase in the expression of immune-related genes [61,62,63]. In order to assess the effect of Yap/Taz inhibitors on inflammatory markers of aging, we compared the expression level of antimicrobial genes (*Attacin-A* (*AttA*), *Diptericin A* (*DptA*), *cecropin A1* (*CecA1*)) between treated and control flies of both sexes at the age of 10 and 20 days.

Three-way ANOVA (gene × concentration × age) showed that there was a significant effect of the gene (*p* < 0.001), a significant effect of the concentration (*p* < 0.05, except males treated with AI and females treated with CD), and a significant effect of the age (*p* < 0.01) of flies (Figure S5, Table S12).

A Duncan test of the 10-day-old control flies versus 20-day-old ones within each substance in male and female flies revealed a significant (*p* < 0.05) age-related increase in the expression level of *AttA* (VP and CD treated males and females, AI females), *DptA* (CD treated males and females, AI females), and *CecA1* (CD and AI treated females) (Figure S5, Tables S12–S14).

A Duncan test of the 20-day-old control flies with increased expression of antimicrobial genes versus age- and sex-matched treated flies within each substance revealed a significant (*p* < 0.05) decrease in the expression level of *AttA* (0.01 μM VP treated males) and *DptA* (0.01 μM VP treated males, 0.1 μM VP treated males and females), demonstrating the anti-aging effect of VP (Figure S5, Tables S12–S14). In addition, a Duncan test of the 10-day-old control flies versus age- and sex-matched treated flies within each substance revealed a significant (*p* < 0.05) anti-inflammatory effect of VP that was manifested in the decreased expression level of *AttA* (0.1 μM VP treated males) and *DptA* (0.1 μM VP treated males and females). At the same time, CD treatment significantly increased the expression of *AttA* (0.1 μM) and *DptA* (0.1 mM and 1 mM) in 20-day-old males, while AI increased the expression of *AttA* (0.1 μM) and *CecA1* (0.1 μM) in females, suggesting that CD and AI do not cause an anti-inflammatory effect (Figure S5, Tables S12–S14).

Thus, the positive effect of YAP/TAZ inhibitors on lifespan was accompanied by both a decrease (VP) and an increase (CD and AI) in the expression levels of inflammatory markers of aging, suggesting a complex relationship between Yap/Taz signaling and the expression rate of the antimicrobial peptide genes.

### 2.4. Effects on Wnt Pathway Activity

Previously, wg was shown to repress the expression of *decapentaplegic* (*dpp*) morphogen via an Armadillo/dTCF/Brinker complex during *Drosophila* leg development [64]. To determine the effect of YAP/TAZ inhibitors on Wnt pathway activity, the expression level of the *dpp* gene was analyzed.

Three-way ANOVA (gene × concentration × age) followed by a post hoc Duncan test revealed a significant (*p* < 0.05) increase in the expression level of *dpp* after ML7 and CD treatment of male flies (Figure S5, Tables S12–S14). The increase in *dpp* expression level caused by treatment with YAP/TAZ inhibitors was consistent with the decrease in *wg* expression level. Moreover, post-developmental RNA interference of *dpp* decreased the mean lifespan of *Drosophila*, demonstrating its pro-longevity potential [65].

## 3. Discussion

Thus, the effects of pharmacological inhibitors of Yki/Yap/Taz on the survival of *D. melanogaster* were investigated in this study. The substances with previously established inhibitor effects on Yap/Taz activity, including Verteporfin (VP) [41,42,43], ML-7 hydrochloride (ML7) [44], Cytochalasin D (CD) [45,46,47], and AICAR (AI) [48] were used.

It has been previously established that evolutionary conservation of both the molecular target and the mechanism of action of a geroprotector increases the probability that the geroprotective effects observed in simpler model organisms will be reproduced in mammals [66]. The functional conservation of the YAP target was confirmed by early studies showing that human YAP is able to rescue the lethality of flies with the Yki mutation [39]. *Drosophila* TEAD/TEF family protein Scalloped (Sc) [67] has been shown to be a DNA-binding transcription factor that partners with Yki to mediate the transcriptional output [68]. The Yki-Sc triggered transcription of target genes has been shown to be conserved in its mammalian homolog [68].

VP has been demonstrated to be a suppressor of Yap-TEAD and Yki-Sd interaction in mammalian and fly cells, respectively [69]. The VP-induced transcriptional changes (decrease in *Diap1* mRNA) in *Drosophila* cells were consistent with the inhibition of the Yki-Sd complex. VP has also been shown to suppress expression of YAP/TAZ transcriptional targets (*SOX2*, *C-MYC*, and *EGFR*) in human patient-derived glioblastoma stem cells and confer significant survival benefit in the *Drosophila* GBM model [70].

Cytoskeletal tension generated through myosin and actin fiber interactions promotes nuclear localization and transcriptional activity of YAP/TAZ [71,72]. ML7 treatment has been shown to decrease cytoskeletal tension [73,74] and inhibit YAP/TAZ through myosin light-chain kinase (MLCK) inhibition [44,72]. Studies in *Drosophila* have also shown that cytoskeletal tension resulted in increased Yki target gene expression [75]. Stretchin-Mlck (Strn-MLCK) is the *Drosophila* ortholog of vertebrate MLCK that shows high sequence homology to the catalytic and regulatory domains of vertebrate MLCK [76,77] and may be suggested as a potential target for ML7.

Similarly, actin polymerization inhibitor CD has also been demonstrated to inhibit YAP/TAZ nuclear accumulation associated with cytoskeletal tension in mammalian and fly models [44,78].

AI-mediated AMPK activation has been shown to inhibit YAP through phosphorylation and stabilization angiomotin-like 1 (AMOTL1), an upstream regulator of YAP [79]. Yki is also regulated by the AMPK in the *Drosophila*, suggesting its inhibition by AI [80].

Thus, the inhibitors used in this study were found to be highly specific for components of the evolutionarily conserved YAP/TAZ pathway.

At the same time, the YAP/TAZ independent effects of these compounds were experimentally confirmed. For example, VP has been demonstrated to promote apoptosis of various tumor cells through increased generation of reactive oxygen radicals, which can result from the inhibition of oxidative phosphorylation [81] and binding of free iron [82,83]. VP can also affect tumor cell proliferation, drug resistance, and tumorigenicity through regulation of the Wnt, PI3K, Ras, mTOR, and NF-κB signaling pathways in various cancer cells [84]. Remarkably, these effects of VP are specific to tumor cells and do not exhibit any toxicity to normal cells, suggesting that VP may have good geroprotective potential.

Previous studies have demonstrated that activation of MLCK increases permeability of vascular [85] and intestinal [86] barriers through myosin light chain (MLC) phosphorylation induced contractility of actomyosin and subsequent weakening of endothelial and epithelial cell–cell adhesion, respectively. It was revealed that the maintenance of the barrier’s integrity is one of the hallmarks of health [87]. Loss of intestinal barrier integrity has been shown to be associated with aging in humans [88] and model organisms [89,90]. At the same time, ML7, a MLCK kinase inhibitor, has been shown to improve vascular endothelial dysfunction in high-fat diet-fed rabbits [91]. It is worth noting that ML7 treatment prevented an age-dependent increase in the level of expression of the antimicrobial peptide gene *DptA* (Figure S5C), suggesting the maintenance of intestinal barrier integrity [62]. It should be taken into account that nonspecific effects of ML7 may also be associated with the inhibition of protein kinase A and protein kinase C, which are involved in a variety of signal transduction pathways [92].

The YAP/TAZ independent effects of CD include modulation of the activity calcium channels [93], and voltage-dependent sodium channels [94], and inhibition of DNA synthesis [95]. Through inhibition of actin polymerization and induction of actin filaments depolymerization, CD also affects the mechanical properties of the cytoskeleton [96] and alters cell motility, adherence, secretion, drug efflux, deformability, morphology, and size [97].

The YAP/TAZ independent effects of AI include stimulation of glucose uptake [98], increase in fatty acid oxidation [99], inhibition of the production of proinflammatory cytokines and mediators [100,101], and activation of antioxidant effects [102]. AI has been demonstrated to inhibit autophagy through the regulation of proteasome activity [103]. Additionally, AI has been found to upregulate autophagy and reduce senescence-associated changes in mesenchymal stromal cells by selectively inhibiting mammalian target of rapamycin complex 1 (mTORC1) in addition to activating AMPK [104]. AI has been demonstrated to be a naturally occurring metabolic intermediate in the biosynthesis of purine nucleotides [105,106], which are essential components for the synthesis of biomolecules essential for cellular survival and proliferation, such as DNA, RNA, ATP, and cofactors for a variety of metabolic and signaling enzymes [107].

The inhibitory effect of used compounds on the expression level of Yki/Yap/Taz target genes in *Drosophila* was analyzed using the qRT-PCR test (Figure 2). It should be noted that among the analyzed Yki target genes (*Diap1*, *dally*, *myc*, *wg*, *CycE*, *vn*), not all of them decreased their expression level in response to treatment with inhibitors. This effect may be associated with non-specific activities and tolerance development in response to long-term (10-day) exposure to inhibitors due to possible activation of negative feedback shunts that foster the activation of Yki target genes regardless of Yki inhibition. The decrease in the effect of inhibitors on the expression of target genes after 20 days of treatment also supports the assumption about the development of tolerance to the action of inhibitors.

The set of repressed genes depended on the used inhibitor and on the sex of the fly. Further research is needed to link the change in fly survival to molecular targets. However, according to the obtained results, the increase in survival was most often associated with a decrease in the level of expression of certain genes, namely *wg* and *E2f1*, that may be associated with aging and longevity (Table 1).

For example, the positive effect on survival time in the case of treatment with VP (males: 0.01 μM and 0.1 μM), CD (males: 1 μM), ML7 (females: 1 μM), and AI (males: 1 μM) was accompanied by a decrease in the expression level of the *wg* gene. On the contrary, an increase in *wg* expression leads to an increase in mortality in ML7 (1 μM) treated males (Table 1).

The *Drosophila wg* gene is the structural homolog of vertebrate *Wnt* genes [108], encoding secreted glycoproteins that act as signaling molecules essential for growth, development, and tissue homeostasis [109,110]. Recently, a new computational approach based on the analysis of networks of co-located loci has identified *wg* among novel genes associated with longevity in *Drosophila* [111]. Experimental data suggest both a positive and a negative role of the Wnt in cell senescence, aging-associated diseases, and organism longevity [112,113]. For example, reduced Wnt signaling has been shown to protect against mutant Huntingtin toxicity in *Drosophila* and prolong the lifespan of flies with Huntington’s disease [114]. In mice, increased age-associated expression of Wnt5a has been correlated with activation of non-canonical Wnt signaling, leading to the aging of hematopoietic stem cells. On the other hand, decreased expression of Wnt5a has been observed to be associated with functional rejuvenation of aged stem-cells [115]. The opposing roles of Wnt ligands mom-2/Wnt (pro-aging) and lin-44/Wnt (anti-aging) have been revealed in *Caenorhabditis elegans* [116]. Meanwhile, studies demonstrating increased Wnt signaling in a Klotho mouse model of accelerated aging [117] and ameliorated aging-related tissue fibrosis after treatment with Wnt inhibitors [118,119] support the pro-aging function of Wnt signaling. Thus, given the dual role of Wnt in aging, the observed mortality-reducing effect may be partly due to its inhibition. We also found that the decrease in wg expression after treatment with YAP/TAZ inhibitors was accompanied by an increase in *dpp* expression (Figure S5), which demonstrated pro-longevity potential in *Drosophila* [65].

In addition, in CD (0.1 and 1 μM) treated males, a decrease in the level of early and late mortality was accompanied by a decrease in the level of *E2f1* expression (Table 1). RNAi mediated knockdown of *E2f1* was found to increase lifespan in *C*. *elegans* by a FOXO/daf-16 mediated mechanism [120].

The process of aging is accompanied by gradual deleterious changes across all levels of biological complexity (molecular, cellular, tissue, and organismal) [121]. Numerous studies support the idea that age-related death of model organisms and flies in particular could be caused by life-limiting pathologies in critical tissues and organs [122,123], such as gut [89,124], fat body [125,126,127,128], muscle [129], brain [130], and neurosecretory cells [131,132,133]. These organs may be considered as potential targets for anti-aging interventions [122].

To investigate whether there are tissue and organ-specific effects of YAP/TAZ inhibitors associated with *wg* and *E2f1* suppression, we used the web application FlyAtlas2 [134]. It should be noted that FlyAtlas2 does not take into account temporal changes in the level of gene expression that occur with age [135,136,137] and therefore has limited application in our study.

It has been found that *wg* is prominently expressed in the salivary glands and eye of both male and female flies. A direct link between Wnt signaling in imago salivary glands and lifespan has not been established yet. However, *wg* was found to downregulate Sunspot protein activity, which transactivates E2f1 and proliferating cell nuclear antigen (PCNA) expression for endoreplication in the salivary gland of *Drosophila* [138]. It has been previously shown that PCNA, which also participates in DNA damage repair [139], is duplicated in the genome of the longest-living mammal bowhead whale (*Balaena mysticetus*). Further investigations should be conducted to determine the localization of *wg* gene expression in adult tissues and to elucidate the contribution of PCNA to *Drosophila* longevity. Thus, the impact of Wnt signaling on the organismal level is likely to be contingent upon a range of variables, such as a tissue-specific environment and genetic background. Nevertheless, the precise role of the Wnt pathway in the positive effect of YAP/TAZ inhibitors has yet to be determined.

The FlyAtlas2 data demonstrate that the *E2f1* gene is expressed in all adult tissues, including those which are associated with age-related functional changes, such as the gut, fat body, muscle, and brain [122]. Overexpression of *dFOXO* in the adult fat body has been demonstrated to increase *Drosophila* lifespan [127,128]. This suggests a potential mechanism of the lifespan-increasing effect of YAP/TAZ inhibition via E2f1-mediated activation of dFOXO in the fat body [120]. Furthermore, E2F1 has been shown to regulate specific metabolic functions in different organs, contributing to global metabolic homeostasis [140]. Additionally, E2F1 activity has been found to be augmented during obesity, potentially contributing to some of the associated comorbidities [140]. Further research is necessary to elucidate the potential role of E2f1 in regulating *Drosophila* lifespan.

The level of transcription of other genes did not show a clear relationship with the survival of the flies (Table 1). In some groups (males and females treated with 1 μM AI, females treated with 1 μM ML), the decrease in mortality was not associated with changes in the expression level of Yki-target genes, suggesting the presence of additional YAP/TAZ-independent activities of these substances.

Indeed, ML7 is widely used as a myosin light-chain kinase (MLCK) inhibitor [141,142]. MLCK is activated by numerous physiological factors and inflammatory or angiogenic mediators, inducing actomyosin contraction and causing endothelial hyperpermeability. Aging is considered as a major risk factor for microvascular dysfunction and hyperpermeability [143]. ML7 has been reported to alleviate advanced glycation end products-induced microvascular hyperpermeability in vivo [144], demonstrating the potential to protect against aging. *Drosophila* suppression of actomyosin contractility via protein kinase A-mediated regulation of MLCK activity has shown to be required for blood–brain barrier integrity [145].

AI is one of the most commonly used pharmacological activators of AMP-activated protein kinase (AMPK) [146]. AMPK is known to control the aging process via an integrated signaling network which affects energy metabolism, autophagic degradation, and stress resistance [147]. Metformin-induced AMPK activation in *C*. *elegans* and mice has been demonstrated to increase lifespan by about 20% and 6%, respectively, in comparison with untreated control animals [148].

In addition, aside from Yap/Taz inhibition through preventing YAP/TAZ-TEAD interaction [149], VP demonstrates antifibrotic effects by reducing the expression of fibrogenic genes in different models [150,151].

Contrarily, CD demonstrated pro-aging properties. CD is known as a potent inhibitor of actin cytoskeleton polymerization [152]. Cytoskeletal integrity was found to be closely related with aging and age-associated diseases [153,154]. Actin polymerization decreases cell stiffness and leads to cellular aging [155], suggesting a possible pro-aging activity of CD.

Despite the finding that *Drosophila* aging is associated with a highly increased expression of antimicrobial peptide genes [61], the results of previous studies on the expression levels of antimicrobial peptide genes after different geroprotective interventions are not unambiguous. While a striking increase in the expression of antimicrobial peptides was observed in glucose-supplemented diet-treated long-lived flies [156], selection for longevity reduced the age-dependent increase in Toll signaling-regulated antimicrobial peptide expression [157]. Knockdown of the Toll receptor [157] or knockdown of individual antimicrobial peptides in the Imd pathway has been shown to extend *Drosophila* lifespan [158]. The overexpression of antimicrobial peptides may contribute to aging [159] or extend lifespan [160] of *Drosophila*, depending on the context of the experiment.

The dual effect of YAP/TAZ inhibitors on the expression of antimicrobial peptide genes (decreased after VP and increased after CD and AI) in long-lived inhibitor-treated flies (Figure S5) demonstrates a complex relationship between antimicrobial peptides and lifespan. In addition, the nonspecific effects of inhibitors on the expression of antimicrobial peptide genes due to the maintenance of intestinal barrier integrity [62] should be considered, as well as the involvement of Yki in Toll receptor-mediated Hippo signaling, which is required for the antimicrobial response triggering antimicrobial peptide production in *Drosophila* [25,161].

Thus, the results of this study indicate that the administration of Yap/Taz inhibitors increased the lifespan of *D. melanogaster*, which was dependent on the sex of the fly and the concentration of the substance. Considering the minimal effect of the inhibitors on the expression levels of Yki target genes and the presence of off-target effects, the lifespan effect cannot be uniquely attributed to the Yki pathway. The analysis of changes in gene expression suggests that suppression of the Wnt and E2f1 signaling pathways may be involved in the observed geroprotective effects. Additional research is necessary to elucidate the relationship between the YAP/TAZ pathway and lifespan.

## 4. Materials and Methods

### 4.1. Drosophila Strain and Experimental Conditions

*Drosophila melanogaster* wild-type *Canton-S* line was obtained from the *Drosophila* Stock Center at Indiana University (Bloomington, IN, USA). To accelerate the aging, the flies were kept at a temperature of 29 °C, which is a common approach in *Drosophila* studies [162]. To maintain constant conditions, Binder KT 115 incubator (Binder, Tuttlingen, Germany) was used. Control end experimental flies were kept on food medium consisting of corn flour—92 g/L, dry yeast—32.1 g/L, agar-agar—5.2 g/L, glucose—136.9 g/L, 8 mL/L—10% solution of Nipagin (methyl 4-hydroxybenzoate, #H5501, Merck, Rahway, NJ, USA) in ethanol, and 5 mL/L of propionic acid (#49685, Merck, Rahway, NJ, USA).

### 4.2. Treatment with Yap/Taz Inhibitors

Verteporfin (VP, #SML0534, Merck, USA), ML-7 hydrochloride (ML7, #I2764, Merck, USA), Cytochalasin D (CD, #C8273, Merck, USA), AICAR (AI, #A9978, Merck, USA) were used as pharmacological inhibitors of Yap/Taz activity. Depending on the solubility of the substances, water, ethanol 96%, or Cyrene (dihydrolevoglucosenone, #807796, Merck, USA) [163] were used as solvents to prepare stock solutions. The stock solutions of VP were prepared with 10% (*v*/*v*) solution of Cyrene in water, ML7—with 50% (*v*/*v*) solution of ethanol in water, CD—with 100% (*v*/*v*) ethanol, and AI—with 100% (*v*/*v*) water.

A volume of 30 μL of stock solutions were pipetted onto the medium surface of each experimental vial. In a control vial, 30 μL of corresponding solvent were pipetted. The final concentrations of substances in the food media were estimated using blue food dye (Brilliant Blue FCF, Roha Dyechem Ltd., Mumbai, India) as a tracer of stock solution diffusion [164]. The obtained results demonstrated a 1:30 dilution of the stock solutions and a final concentration of VP—0.01, 0.1, 1, 10 μM, ML7—0.01, 0.1, 1 μM, CD, and AI—0.1, 1 μM.

Treatment with inhibitors was started from the first day of imago life and continued for 10 days for analysis of survival throughout the lifetime for qRT-PCR assay.

### 4.3. RNA Isolation and Real-Time Quantitative RT-PCR

The expression level of Yki and its target genes (Death-associated inhibitor of apoptosis 1 (Diap1), division abnormally delayed (dally), Myc (myc), wingless (wg), Cyclin E (CycE), vein (vn), and E2F transcription factor 1 (E2f1)) [165,166], immune-related genes (Attacin-A (AttA) Cecropin A1 (CecA1), Diptericin A (DptA1) [61], and target gene repressed by wg signaling (decapentaplegic (*dpp*)) [64,167] was measured by the real-time quantitative reverse transcription-polymerase chain reaction (qRT-PCR). RNA was isolated using an Aurum Total RNA Mini kit (Bio-Rad, Hercules, CA, USA) according to the manufacturer’s instructions. RNA concentration was measured using a Quant-iT RNA Assay Kit (Invitrogen, Waltham, MA, USA) according to the manufacturer’s instructions. cDNA was synthesized according to the iScript cDNA Synthesis Kit (Bio-Rad, Hercules, CA, USA) from the resulting RNA solution. The reaction mixture for the PCR reaction was prepared based on qPCR mix-HS SYBR (Evrogen, Moscow, Russia) and primers (Table S15). The primer design was performed using the QuantPrime online tool [168]. The polymerase chain reaction was carried out in a CFX96 amplifier (Bio-Rad, Hercules, CA, USA) using the following program: (1) 95 °C for 30 s, (2) 95 °C for 10 s, (3) 60 °C for 30 s, (4) steps 2–3 were repeated 49 times, (5) DNA melting step.

The expression of the studied genes was calculated relative to the expression of the reference genes *β-Tubulin at 56D* (*β-Tubulin*) and *Ribosomal protein L32* (*RpL32*) using threshold cycles (Cts). The delta Ct (dCt) values were calculated as the differences in Ct values for target genes and reference genes. The values of Ct were taken from the CFX Manager 3.1 software (Bio-Rad, Hercules, CA, USA). For each experimental group, 20 males and 10 females were used. The experiments were carried out in three biological replicates with three technical replicates in each.

### 4.4. Analysis of Survival

Survival was assessed using the concentrations of substances affecting the expression levels of Yki target genes. The emerged imagoes were separated by sex (mated males and non-virgin females) and transferred to control and experimental vials within 24 h. Flies were maintained at 29 °C. The dead individuals were recorded daily. The median and maximum (age of 90% mortality) lifespan were calculated and survival curves were plotted. The experiments were performed separately for each sex in two biological replicates (30 flies per vial, 5 vials, 150 flies per group per replicate).

### 4.5. Statistical Analysis

To determine the statistical significance of differences in the levels of gene expression, multi-factor analysis of variance (ANOVA) with post hoc Duncan’s multiple-range test were used [169]. The survival curves were created using the Kaplan–Meier method [170]. The statistical significance of differences between survival curves was evaluated using the log-rank test [171]. A Fleming–Harrington test was used to estimate differences between control and experimental groups in earlier or later deaths [172] The significance of differences in median maximum lifespan was assessed using Fisher’s exact test [173]. Bonferroni correction was used to adjust for multiple comparisons. Statistical data analysis was performed using the TIBCO Statistica, version 13.3 (TIBCO Software, Palo Alto, CA, USA) and the online application for survival analysis OASIS 2 [174].

## Figures and Tables

**Table 1:** Overall effects of *Drosophila* treatment with Yap/Taz inhibitors for 10 days.

Inhibitor	C (μM)	Sex	Gene Expression								Mortality	
			*CycE*	*dally*	*Diap1*	*E2f1*	*myc*	*vn*	*wg*	*yki*	Early	Late
VP	0.01	♂	↑	0	0	0	↑	↑	↓	↑	↓	↓
	0.1	♂	0	0	0	0	↑	↑	↓	0	0	↓
ML	0.1	♂	0	0	0	0	0	0	↓	0	0	0
	1	♂	0	0	0	0	0	0	↑	0	0	↑
CD	0.1	♂	0	0	0	↓	0	0	0	0	↓	↓
	1	♂	0	0	↓	↓	0	0	↓	↓	↓	↓
AI	0.1	♂	↑	0	0	0	0	0	↓	0	↓	↓
	1	♂	0	0	0	0	0	0	0	0	↓	↓
VP	0.01	♀	↓	0	0	0	0	0	0	0	0	0
	0.1	♀	0	0	0	0	0	0	0	0	0	0
ML	0.1	♀	0	0	0	0	0	0	0	0	0	↓
	1	♀	0	0	0	0	0	0	↓	0	0	↓
CD	0.1	♀	↑	0	0	0	↑	0	↓	0	↑	0
	1	♀	0	0	0	0	0	0	↓	0	0	0
AI	0.1	♀	0	0	0	0	0	0	↓	↑	0	0
	1	♀	0	0	0	0	0	0	0	↑	↓	↓

C (μM)—concentration; Sex: ♂—male; ♀—female; Effects: 0—no significant difference, ↑—increase, ↓—decrease.

## Data Availability

All data generated or analyzed during this study are included in this published article.

## Supplementary Materials

The following supporting information can be downloaded at https://www.mdpi.com/article/10.3390/ijms24066006/s1.
