# Supplementary material for: Studying the Geroprotective Properties of YAP/TAZ Signaling Inhibitors on Drosophila melanogaster Model
Source: Int J Mol Sci. 2023 Mar 22;24(6):6006. doi: 10.3390/ijms24066006 (PMC10058302; doi:10.3390/ijms24066006)
Supplement: Supplementary file 1 [file ijms-24-06006-s001.zip › Table S5.pdf]

Table S5. The expression level of Yki target genes in 10 day-old females after treatment with YAP/TAZ inhibitors.

Gene	Concentration (μM)	Mean (dCt)	SE	DT (p)
Verteporfin				
CycE	0 (control)	7.50	0.10	
	0.01	7.97	0.06	0.0408↓
	0.1	7.71	0.07	0.3040
	1	7.72	0.09	0.3107
	10	7.73	0.15	0.3204
dally	0 (control)	7.03	0.04	
	0.01	7.11	0.35	0.7269
	0.1	7.20	0.26	0.4485
	1	7.26	0.11	0.3298
	10	6.74	0.12	0.1627
Diap1	0 (control)	4.88	0.04	
	0.01	4.63	0.09	0.2774
	0.1	4.82	0.07	0.7818
	1	5.24	0.19	0.1175
	10	4.63	0.11	0.2824
E2f1	0 (control)	5.72	0.09	
	0.01	5.86	0.22	0.5748
	0.1	5.75	0.18	0.9077
	1	5.80	0.12	0.7472
	10	5.53	0.14	0.4024
myc	0 (control)	5.70	0.13	
	0.01	5.46	0.07	0.3210
	0.1	5.26	0.04	0.0669
	1	6.17	0.16	0.0529
	10	5.48	0.12	0.3531
vn	0 (control)	8.81	0.17	
	0.01	8.65	0.06	0.4607
	0.1	8.85	0.06	0.8419
	1	9.40	0.24	0.0060↓
	10	8.30	0.09	0.0207↑
wg	0 (control)	12.52	0.22	
	0.01	12.52	0.10	0.9954
	0.1	12.65	0.16	0.5672
	1	12.05	0.23	0.0251↑
	10	12.05	0.25	0.0304↑
Yki	0 (control)	5.55	0.10	
	0.01	5.28	0.12	0.2627
	0.1	5.14	0.06	0.0957
	1	5.77	0.14	0.3507
	10	4.96	0.12	0.0140↑
ML-7				
CycE	0 (control)	8.70	0.28	

	0.01	7.40	0.15	0.0052↑
	0.1	8.71	0.38	0.9866
	1	8.16	0.26	0.2399
dally	0 (control)	7.86	0.11	
	0.01	8.05	0.41	0.6666
	0.1	7.97	0.26	0.7881
	1	8.80	0.28	0.0541
Diap1	0 (control)	4.90	0.37	
	0.01	4.89	0.31	0.9880
	0.1	4.41	0.14	0.2703
	1	5.48	0.29	0.2219
E2f1	0 (control)	5.34	0.13	
	0.01	5.39	0.21	0.9180
	0.1	5.69	0.27	0.4903
	1	5.55	0.14	0.6708
myc	0 (control)	6.90	0.27	
	0.01	6.17	0.14	0.0980
	0.1	7.05	0.44	0.7110
	1	6.59	0.20	0.4521
vn	0 (control)	9.53	0.22	
	0.01	9.21	0.26	0.4765
	0.1	9.10	0.13	0.3517
	1	8.93	0.15	0.2065
wg	0 (control)	8.53	0.74	
	0.01	8.47	0.57	0.8777
	0.1	9.26	0.38	0.1388
	1	10.10	0.19	0.0007↓
Yki	0 (control)	5.61	0.16	
	0.01	5.50	0.10	0.8143
	0.1	5.26	0.21	0.4959
	1	5.59	0.12	0.9623
Cytochalasin D				
CycE	0 (control)	9.57	0.16	
	0.1	8.88	0.22	0.0047↑
	1	9.18	0.25	0.0794
dally	0 (control)	8.31	0.12	
	0.1	7.71	0.17	0.0095
	1	8.07	0.28	0.2676
Diap1	0 (control)	4.86	0.04	
	0.1	4.52	0.03	0.2021
	1	4.59	0.11	0.3039
E2f1	0 (control)	4.84	0.08	
	0.1	4.78	0.07	0.7948
	1	4.65	0.09	0.4453
myc	0 (control)	6.38	0.05	
	0.1	5.87	0.10	0.0293↑
	1	6.17	0.19	0.3402
vn	0 (control)	9.03	0.06	

	0.1	8.93	0.07	0.6429
	1	9.05	0.20	0.9327
wg	0 (control)	9.69	0.29	
	0.1	10.14	0.34	0.0467↓
	1	10.31	0.09	0.0074↓
Yki	0 (control)	5.04	0.05	
	0.1	4.86	0.05	0.4339
	1	4.80	0.12	0.3543
AICAR				
CycE	0 (control)	9.23	0.08	
	0.1	8.74	0.29	0.1172
	1	9.25	0.11	0.9329
dally	0 (control)	8.00	0.07	
	0.1	8.08	0.13	0.7739
	1	8.41	0.06	0.1557
Diap1	0 (control)	4.28	0.06	
	0.1	4.09	0.09	0.5081
	1	4.34	0.09	0.8251
E2f1	0 (control)	4.67	0.07	
	0.1	4.09	0.08	0.0664
	1	4.38	0.08	0.2921
myc	0 (control)	5.80	0.09	
	0.1	5.37	0.10	0.1534
	1	5.76	0.04	0.8911
vn	0 (control)	8.95	0.04	
	0.1	8.88	0.11	0.8045
	1	9.28	0.09	0.2980
wg	0 (control)	9.01	0.57	
	0.1	9.85	0.27	0.0050↓
	1	8.58	0.52	0.1701
Yki	0 (control)	5.47	0.06	
	0.1	4.83	0.11	0.0262↑
	1	4.79	0.15	0.0230↑

dCt – delta Ct (cycle thresholds) values were calculated as the differences in Ct values for target genes and reference genes (*β-Tubulin* and *RpL32*). The Ct values are inversely proportional to the mRNA transcript levels. Higher dCt values represent a lower expression level. SE – standard error of mean, p – p-value. Significance was determined by two-way ANOVA followed by post-hoc Duncan test (DT), ↑ – increase, ↓ – decrease.
