# Supplementary material for: Studying the Geroprotective Properties of YAP/TAZ Signaling Inhibitors on Drosophila melanogaster Model
Source: Int J Mol Sci. 2023 Mar 22;24(6):6006. doi: 10.3390/ijms24066006 (PMC10058302; doi:10.3390/ijms24066006)
Supplement: Supplementary file 1 [file ijms-24-06006-s001.zip › Figure S1.pdf]

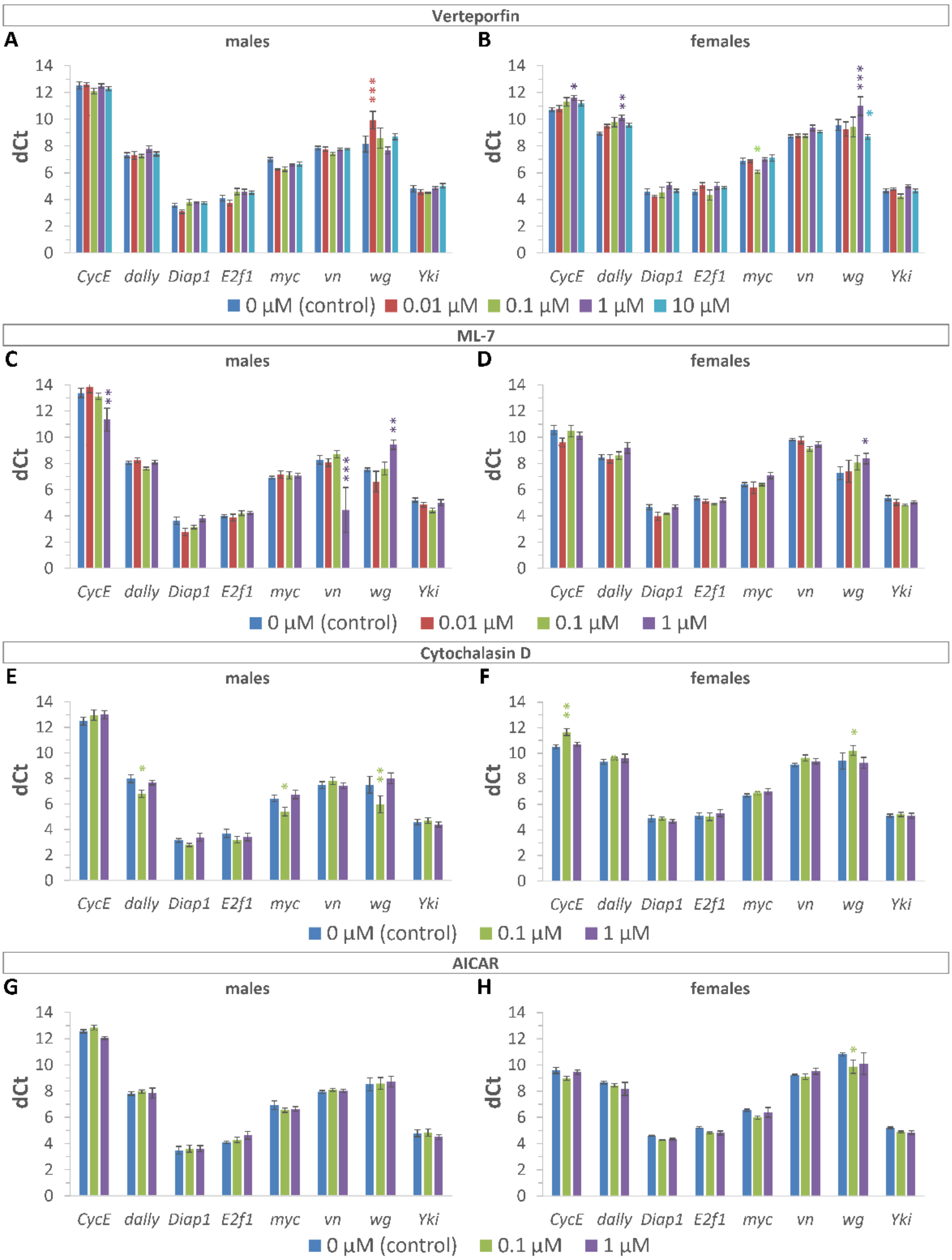

Figure S1. Effects of YAP/TAZ inhibitors on the expression level of Yki target genes. Estimation of the expression level in 20 day-old males and females after treatment with VP (**A**, **B**), ML7 (**C**, **D**), CD (**E**, **F**), AI (**G**, **H**). Two-way ANOVA (age × gene) followed by post hoc Duncan test was used to compare differences in expression levels of genes between 10-day-old and 20-day-old flies, *$p<0.05$, **$p<0.01$, ***$p<0.001$. The Ct (cycle thresholds) values are inversely proportional to the mRNA transcript levels. The delta Ct (dCt) values were calculated as the differences in Ct values for target genes and reference genes (*β-Tubulin* and *RpL32*). Higher dCt values represent a lower expression level. The error bars show standard errors. The experiments were performed in three biological replicates and three technical replicates (n = 3, repeated three times). The samples included 20 males and 10 females.
