# Supplementary material for: Studying the Geroprotective Properties of YAP/TAZ Signaling Inhibitors on Drosophila melanogaster Model
Source: Int J Mol Sci. 2023 Mar 22;24(6):6006. doi: 10.3390/ijms24066006 (PMC10058302; doi:10.3390/ijms24066006)
Supplement: Supplementary file 1 [file ijms-24-06006-s001.zip › Figure S2.pdf]

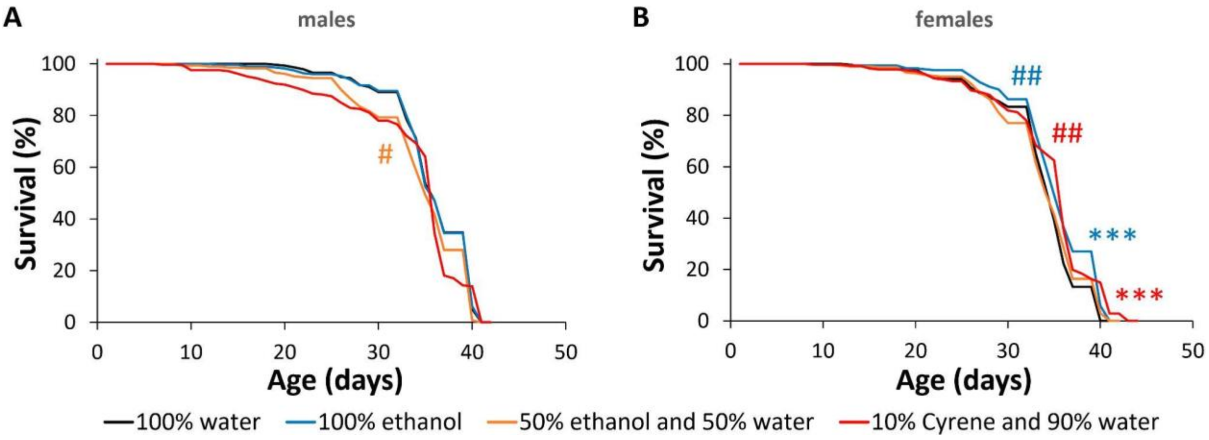

Figure S2. Survival curves of male (**A**) and female (**B**) flies of control groups maintained on different solvents. Fleming-Harrington test sensitive against early (#p<0.05, ##p<0.01, ###p<0.001) and later (*p<0.05, **p<0.01, ***p<0.001) differences were used to compare survival curves of flies treated with water solvent versus flies treated with ethanol and Cyrene based solvents. Bonferroni correction was used for multiple comparisons. The results of two independent experiments are presented; n = 293 (100% water, males); n = 278 (100% ethanol, males); n = 290 (50% ethanol, males); n = 287 (10% Cyrene, males); n = 323 (100% water, females); n = 292 (100% ethanol, females); n = 300 (50% ethanol, females); n = 282 (10% Cyrene, females).
