# Supplementary material for: Studying the Geroprotective Properties of YAP/TAZ Signaling Inhibitors on Drosophila melanogaster Model
Source: Int J Mol Sci. 2023 Mar 22;24(6):6006. doi: 10.3390/ijms24066006 (PMC10058302; doi:10.3390/ijms24066006)
Supplement: Supplementary file 1 [file ijms-24-06006-s001.zip › Figure S3.pdf]

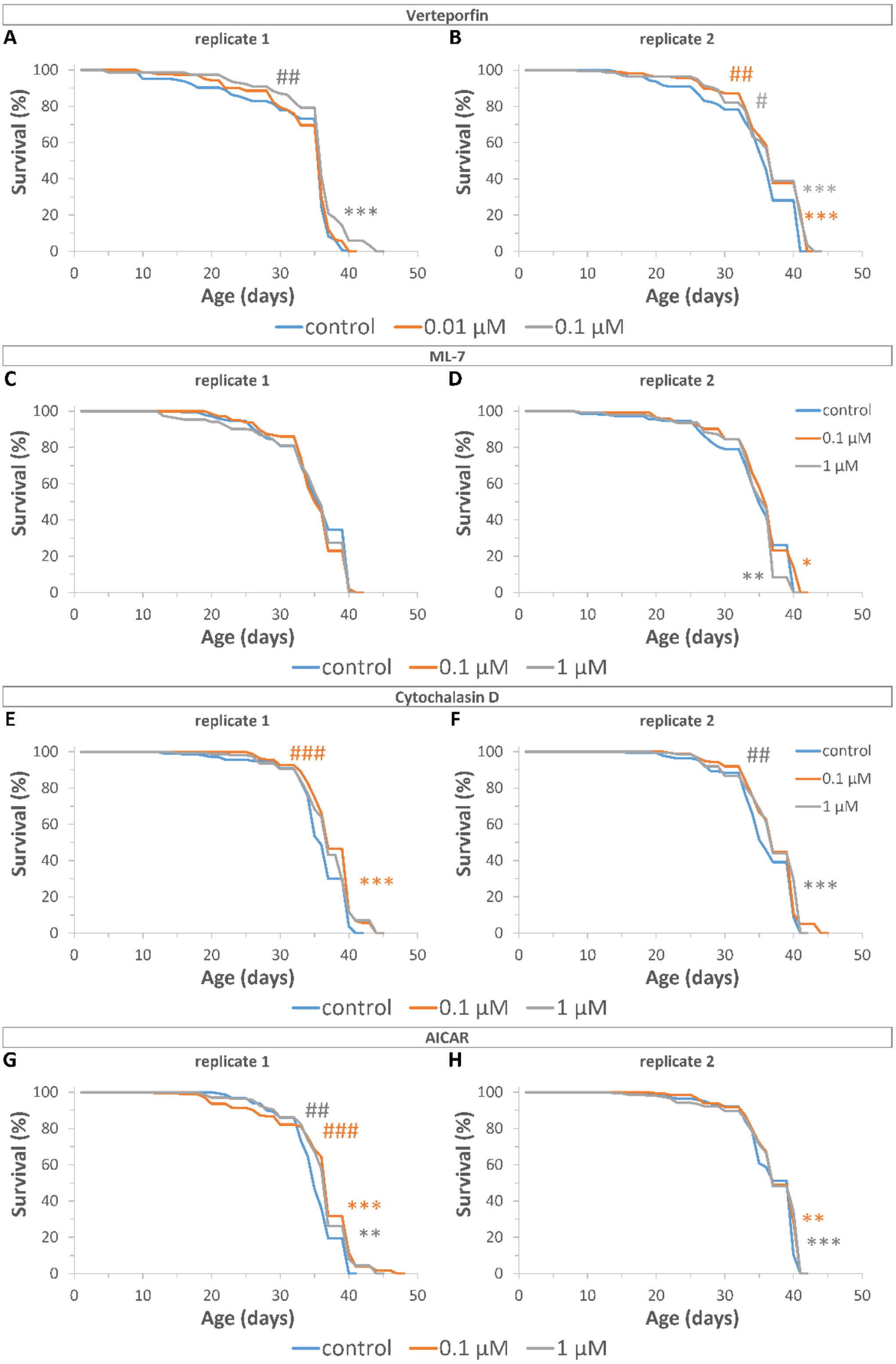

Figure S3. Effects of YAP/TAZ inhibitors on the lifespan of males. The results of two independent replicate experiments after treatment with VP (**A**, **B**), ML7 (**C**, **D**), CD (**E**, **F**), AI (**G**, **H**) are presented. Fleming-Harrington test sensitive against early ($^{\#}p<0.05$, $^{\#\#}p<0.01$, $^{\#\#\#}p<0.001$) and later (*p<0.05, **p<0.01, ***p<0.001) differences were used to compare survival curves of inhibitor-treated and control flies. Bonferroni correction was used for multiple comparisons. Two independent experiments were performed; n = 145 (VP control, males, replicate 1); n = 141 (0.01 μM VP, males, replicate 1); n = 154 (0.1 μM VP, males, replicate 1); n = 147 (ML control, males, replicate 1); n = 144 (0.1 μM ML, males, replicate

1); n = 153 (1 μM ML, males, replicate 1); n = 140 (CD control, males, replicate 1); n = 163 (0.1 μM CD, males, replicate 1); n = 153 (1 μM CD, males, replicate 1); n = 150 (AI control, males, replicate 1); n = 174 (0.1 μM AI, males, replicate 1); n = 157 (1 μM AI, males, replicate 1); n = 152 (VP control, males, replicate 2); n = 165 (0.01 μM VP, males, replicate 2); n = 159 (0.1 μM VP, males, replicate 2); n = 153 (ML control, males, replicate 2); n = 143 (0.1 μM ML, males, replicate 2); n = 156 (1 μM ML, males, replicate 2); n = 150 (CD control, males, replicate 2); n = 161 (0.1 μM CD, males, replicate 2); n = 150 (1 μM CD, males, replicate 2); n = 143 (AI control, males, replicate 2); n = 147 (0.1 μM AI, males, replicate 2); n = 156 (1 μM AI, males, replicate 2).
