# Supplementary material for: Studying the Geroprotective Properties of YAP/TAZ Signaling Inhibitors on Drosophila melanogaster Model
Source: Int J Mol Sci. 2023 Mar 22;24(6):6006. doi: 10.3390/ijms24066006 (PMC10058302; doi:10.3390/ijms24066006)
Supplement: Supplementary file 1 [file ijms-24-06006-s001.zip › Figure S4.pdf]

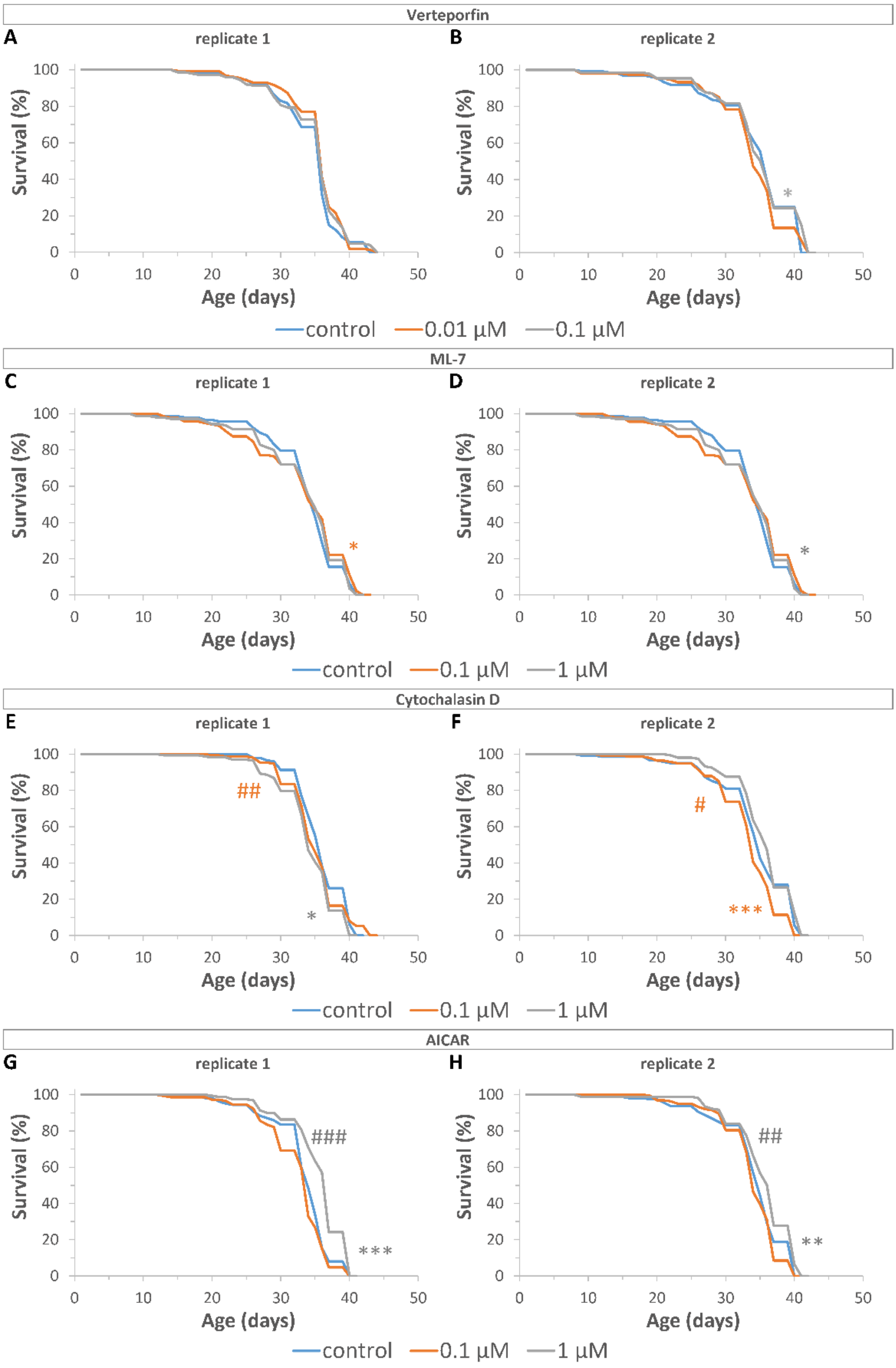

Figure S4. Effects of YAP/TAZ inhibitors on the lifespan of females. The results of two independent replicate experiments after treatment with VP (**A**, **B**), ML7 (**C**, **D**), CD (**E**, **F**), AI (**G**, **H**) are presented. Fleming-Harrington test sensitive against early ($^{\#}$p<0.05, $^{\#\#}$p<0.01, $^{\#\#\#}$p<0.001) and later (*p<0.05, **p<0.01, ***p<0.001) differences were used to compare survival curves of inhibitor-treated and control flies. Bonferroni correction was used for multiple comparisons. Two independent experiments were performed; n = 147 (VP control, females, replicate 1); n = 157 (0.01 µM VP, females, replicate 1); n = 150 (0.1 µM VP, females, replicate 1); n = 142 (ML control, females, replicate 1); n = 136 (0.1 µM ML, females, replicate

1); n = 140 (1 μM ML, females, replicate 1); n = 149 (CD control, females, replicate 1); n = 152 (0.1 μM CD, females, replicate 1); n = 168 (1 μM CD, females, replicate 1); n = 163 (AI control, females, replicate 1); n = 146 (0.1 μM AI, females, replicate 1); n = 161 (1 μM AI, females, replicate 1); n = 145 (VP control, females, replicate 2); n = 148 (0.01 μM VP, females, replicate 2); n = 131 (0.1 μM VP, females, replicate 2); n = 158 (ML control, females, replicate 2); n = 138 (0.1 μM ML, females, replicate 2); n = 143 (1 μM ML, females, replicate 2); n = 143 (CD control, females, replicate 2); n = 141 (0.1 μM CD, females, replicate 2); n = 162 (1 μM CD, females, replicate 2); n = 160 (AI control, females, replicate 2); n = 164 (0.1 μM AI, females, replicate 2); n = 169 (1 μM AI, females, replicate 2).
