# Supplementary material for: Studying the Geroprotective Properties of YAP/TAZ Signaling Inhibitors on Drosophila melanogaster Model
Source: Int J Mol Sci. 2023 Mar 22;24(6):6006. doi: 10.3390/ijms24066006 (PMC10058302; doi:10.3390/ijms24066006)
Supplement: Supplementary file 1 [file ijms-24-06006-s001.zip › Table S1.pdf]

Table S1. Age-related changes in the expression level of Yki target genes.

Gene	Sex	Age (days)	Mean (dCt)	Standard Error	p-value
CycE	♂	10	11.6	0.2	
	♂	20	12.7	0.1	0.0001
dally	♂	10	7.2	0.2	
	♂	20	7.8	0.1	0.0409
Diap1	♂	10	2.8	0.2	
	♂	20	3.4	0.1	0.0316
E2f1	♂	10	4.3	0.1	
	♂	20	4.0	0.1	0.2499
myc	♂	10	7.2	0.3	
	♂	20	6.8	0.1	0.2122
vn	♂	10	8.2	0.3	
	♂	20	7.9	0.1	0.2864
wg	♂	10	7.5	0.4	
	♂	20	7.9	0.3	0.1902
Yki	♂	10	4.9	0.2	
	♂	20	4.8	0.1	0.7089
CycE	♀	10	8.7	0.2	
	♀	20	10.3	0.1	0.0000
dally	♀	10	7.8	0.1	
	♀	20	8.8	0.1	0.0000
Diap1	♀	10	4.7	0.1	
	♀	20	4.7	0.1	0.8341
E2f1	♀	10	5.1	0.1	
	♀	20	5.1	0.1	0.7278
myc	♀	10	6.2	0.1	
	♀	20	6.6	0.1	0.0373
vn	♀	10	9.1	0.1	
	♀	20	9.2	0.1	0.5022
wg	♀	10	9.9	0.4	
	♀	20	9.3	0.3	0.0013
Yki	♀	10	5.4	0.1	
	♀	20	5.1	0.1	0.1415

♂ – males, ♀ – females; dCt – delta Ct (cycle thresholds) values were calculated as the differences in Ct values for target genes and reference genes (*β-Tubulin* and *RpL32*). The Ct values are inversely proportional to the mRNA transcript levels. Higher dCt values represent a lower expression level. p-value – significance was determined by two-way ANOVA followed by post-hoc Duncan test.
