# Supplementary material for: Studying the Geroprotective Properties of YAP/TAZ Signaling Inhibitors on Drosophila melanogaster Model
Source: Int J Mol Sci. 2023 Mar 22;24(6):6006. doi: 10.3390/ijms24066006 (PMC10058302; doi:10.3390/ijms24066006)
Supplement: Supplementary file 1 [file ijms-24-06006-s001.zip › Table S2.pdf]

Table S2. Two-way ANOVA of age-related changes in the expression level of Yki target genes in male and female flies.

Sex	Source of variation	SS	DF	MS	F	p
♂	Gene	4066.12	7	580.87	403.97	0.0000
	Age	5.86	1	5.86	4.07	0.0440
	Gene × Age	38.66	7	5.52	3.84	0.0004
	Error	795.16	553	1.44		
♀	Gene	2253.38	7	321.91	400.85	0.0000
	Age	9.75	1	9.75	12.14	0.0005
	Gene × Age	69.69	7	9.96	12.40	0.0000
	Error	448.92	559	0.80		

♂ – male; ♀ – female; SS – sum-of-squares; DF – degrees of freedom; MS – mean squares. Results of F-tests: F – F-value and p – p-value.
