# Supplementary material for: Studying the Geroprotective Properties of YAP/TAZ Signaling Inhibitors on Drosophila melanogaster Model
Source: Int J Mol Sci. 2023 Mar 22;24(6):6006. doi: 10.3390/ijms24066006 (PMC10058302; doi:10.3390/ijms24066006)
Supplement: Supplementary file 1 [file ijms-24-06006-s001.zip › Table S3.pdf]

Table S3. Two-way ANOVA of the effect of Yap/Taz inhibitors on the expression level of Yki target genes in 10-day-old male and female flies.

Substance	Sex	Source of variation	SS	DF	MS	F	p
VP	♂	Gene	1446.39	7	206.63	105.601	0.000000
		Concentration	29.01	4	7.25	3.706	0.005773
		Gene × Concentration	118.59	28	4.24	2.164	0.000798
		Error	620.27	317	1.96		
ML7	♂	Gene	2067.13	7	295.30	553.90	0.000000
		Concentration	5.52	3	1.84	3.45	0.017177
		Gene × Concentration	26.44	21	1.26	2.36	0.000954
		Error	134.88	253	0.53		
CD	♂	Gene	1437.930	7	205.419	771.26	0.000000
		Concentration	6.213	2	3.106	11.66	0.000017
		Gene × Concentration	4.579	14	0.327	1.23	0.257662
		Error	50.605	190	0.266		
AI	♂	Gene	1451.440	7	207.349	433.02	0.000000
		Concentration	1.564	2	0.782	1.63	0.197951
		Gene × Concentration	54.479	14	3.891	8.13	0.000000
		Error	91.937	192	0.479		
VP	♀	Gene	1913.58	7	273.37	1398.06	0.000000
		Concentration	9.00	4	2.25	11.51	0.000000
		Gene × Concentration	12.73	28	0.45	2.33	0.000253
		Error	62.18	318	0.20		
ML7	♀	Gene	743.67	7	106.24	139.15	0.000000
		Concentration	9.41	3	3.14	4.11	0.007175
		Gene × Concentration	33.31	21	1.59	2.08	0.004550
		Error	192.40	252	0.76		
CD	♀	Gene	927.41	7	132.49	604.43	0.000000
		Concentration	2.29	2	1.14	5.22	0.006225
		Gene × Concentration	5.50	14	0.39	1.79	0.042299
		Error	41.21	188	0.22		
AI	♀	Gene	922.43	7	131.78	385.59	0.000000
		Concentration	1.24	2	0.62	1.81	0.166154
		Gene × Concentration	14.95	14	1.07	3.13	0.000205
		Error	65.27	191	0.34		

VP – Verteporfin; ML7 – ML-7 hydrochloride; CD – Cytochalasin D; AI – AICAR; ♂ – male; ♀ – female; SS – sum-of-squares; DF – degrees of freedom; MS – mean squares. Results of F-tests: F – F-value and p – p-value.
