# Supplementary material for: Studying the Geroprotective Properties of YAP/TAZ Signaling Inhibitors on Drosophila melanogaster Model
Source: Int J Mol Sci. 2023 Mar 22;24(6):6006. doi: 10.3390/ijms24066006 (PMC10058302; doi:10.3390/ijms24066006)
Supplement: Supplementary file 1 [file ijms-24-06006-s001.zip › Table S4.pdf]

Table S4. The expression level of Yki target genes in 10 day-old males after treatment with YAP/TAZ inhibitors.

Gene	Concentration (μM)	Mean (dCt)	SE	DT (p)
Verteporfin				
CycE	0 (control)	10.35	0.52	
	0.01	8.52	0.26	0.0168 ↑
	0.1	9.24	0.36	0.1458
	1	9.38	0.29	0.1836
	10	9.35	0.11	0.1845
dally	0 (control)	6.73	0.84	
	0.01	5.43	0.13	0.1050
	0.1	6.17	0.25	0.3989
	1	5.84	0.30	0.2254
	10	5.82	0.28	0.2398
Diap1	0 (control)	2.15	0.53	
	0.01	2.86	0.11	0.2878
	0.1	3.44	0.27	0.0752
	1	3.83	0.25	0.0215 ↓
	10	3.40	0.16	0.0747
E2f1	0 (control)	4.89	0.40	
	0.01	4.07	0.13	0.2997
	0.1	4.66	0.20	0.7210
	1	4.47	0.13	0.5699
	10	4.37	0.07	0.4953
myc	0 (control)	7.49	1.28	
	0.01	5.68	0.17	0.0235 ↑
	0.1	5.95	0.41	0.0484 ↑
	1	5.75	0.13	0.0293 ↑
	10	5.83	0.12	0.0351 ↑
vn	0 (control)	8.93	1.07	
	0.01	6.76	0.17	0.0041 ↑
	0.1	7.39	0.31	0.0398 ↑
	1	7.12	0.13	0.0161 ↑
	10	6.93	0.15	0.0076 ↑
wg	0 (control)	6.90	1.62	
	0.01	8.44	0.48	0.0409 ↓
	0.1	8.82	0.28	0.0110 ↓
	1	9.55	0.23	0.0004 ↓
	10	9.69	0.31	0.0002 ↓
Yki	0 (control)	5.68	0.67	
	0.01	4.31	0.12	0.0847 ↑
	0.1	4.96	0.39	0.3304
	1	4.55	0.23	0.1439
	10	4.18	0.09	0.0590 ↑
ML-7				
CycE	0 (control)	12.22	0.15	
	0.01	13.01	0.36	0.0353 ↓
	0.1	12.49	0.32	0.4322
	1	12.81	0.37	0.1086
dally	0 (control)	7.13	0.09	
	0.01	7.69	0.36	0.1570
	0.1	7.27	0.17	0.7088
	1	7.80	0.43	0.0931

Diap1	0 (control)	3.47	0.24	
	0.01	3.84	0.39	0.2941
	0.1	3.34	0.11	0.7042
	1	3.99	0.24	0.1612
E2f1	0 (control)	4.47	0.14	
	0.01	4.64	0.18	0.6414
	0.1	4.44	0.13	0.9429
	1	4.53	0.10	0.8509
myc	0 (control)	7.10	0.20	
	0.01	7.49	0.32	0.3394
	0.1	7.64	0.28	0.1867
	1	7.12	0.18	0.9681
vn	0 (control)	7.96	0.15	
	0.01	8.43	0.19	0.2304
	0.1	8.54	0.22	0.1432
	1	8.19	0.14	0.5252
wg	0 (control)	8.36	0.28	
	0.01	7.98	0.43	0.3123
	0.1	9.22	0.15	0.0206 ↓
	1	7.24	0.25	0.0049 ↑
Yki	0 (control)	4.75	0.10	
	0.01	5.26	0.18	0.1833
	0.1	5.00	0.10	0.4735
	1	5.12	0.11	0.3197
Cytochalasin D				
CycE	0 (control)	11.94	0.15	
	0.1	11.73	0.13	0.4165
	1	11.90	0.26	0.8656
dally	0 (control)	7.48	0.09	
	0.1	7.13	0.12	0.1922
	1	7.35	0.03	0.5942
Diap1	0 (control)	2.87	0.11	
	0.1	3.24	0.05	0.1343
	1	3.46	0.07	0.0215 ↓
E2f1	0 (control)	3.71	0.08	
	0.1	4.19	0.13	0.0488 ↓
	1	4.56	0.22	0.0007 ↓
myc	0 (control)	6.87	0.10	
	0.1	6.97	0.12	0.6707
	1	7.17	0.08	0.2607
vn	0 (control)	7.69	0.12	
	0.1	7.85	0.07	0.5200
	1	8.11	0.09	0.1089
wg	0 (control)	8.16	0.17	
	0.1	8.42	0.24	0.2737
	1	8.78	0.32	0.0145 ↓
Yki	0 (control)	4.60	0.13	
	0.1	4.58	0.08	0.9189
	1	5.20	0.47	0.0147 ↓
AICAR				
CycE	0 (control)	11.93	0.19	
	0.1	11.12	0.31	0.0176 ↑
	1	11.68	0.18	0.4434
dally	0 (control)	7.32	0.07	
	0.1	7.02	0.09	0.3967
	1	7.37	0.14	0.8627

Diap1	0 (control)	2.92	0.04	
	0.1	2.76	0.06	0.6380
	1	2.88	0.11	0.8911
E2f1	0 (control)	4.10	0.03	
	0.1	3.90	0.05	0.5563
	1	4.00	0.11	0.7535
myc	0 (control)	7.27	0.11	
	0.1	6.65	0.12	0.0975
	1	6.93	0.11	0.3179
vn	0 (control)	8.24	0.14	
	0.1	7.68	0.07	0.1032
	1	7.78	0.20	0.1555
wg	0 (control)	6.77	0.18	
	0.1	9.64	0.05	0.0000 ↓
	1	6.88	0.93	0.7520
Yki	0 (control)	4.73	0.14	
	0.1	4.34	0.13	0.2263
	1	4.25	0.09	0.1649

dCt – delta Ct (cycle thresholds) values were calculated as the differences in Ct values for target genes and reference genes (*β-Tubulin* and *RpL32*). The Ct values are inversely proportional to the mRNA transcript levels. Higher dCt values represent a lower expression level. SE – standard error of mean, p – p-value. Significance was determined by two-way ANOVA followed by post-hoc Duncan test (DT), ↑ – increase, ↓ – decrease.
