# Supplementary material for: Studying the Geroprotective Properties of YAP/TAZ Signaling Inhibitors on Drosophila melanogaster Model
Source: Int J Mol Sci. 2023 Mar 22;24(6):6006. doi: 10.3390/ijms24066006 (PMC10058302; doi:10.3390/ijms24066006)
Supplement: Supplementary file 1 [file ijms-24-06006-s001.zip › Table S6.pdf]

Table S6. Two-way ANOVA of the effect of Yap/Taz inhibitors on the expression level of Yki target genes in 20-day-old male and female flies.

Substance	Sex	Source of variation	SS	DF	MS	F	p
VP	♂	Gene	2500.13	7	357.16	645.18	0.000000
		Concentration	1.43	4	0.36	0.64	0.631395
		Gene × Concentration	41.82	28	1.49	2.70	0.000016
		Error	176.04	318	0.55		
ML7	♂	Gene	2223.18	7	317.60	183.427	0.000000
		Concentration	7.34	3	2.45	1.413	0.239536
		Gene × Concentration	177.42	21	8.45	4.880	0.000000
		Error	436.33	252	1.73		
CD	♂	Gene	1818.815	7	259.831	261.428	0.000000
		Concentration	12.744	2	6.372	6.411	0.002017
		Gene × Concentration	28.694	14	2.050	2.062	0.015491
		Error	190.827	192	0.994		
AI	♂	Gene	1586.51	7	226.64	377.81	0.000000
		Concentration	0.30	2	0.15	0.25	0.777778
		Gene × Concentration	5.63	14	0.40	0.67	0.801010
		Error	114.58	191	0.60		
VP	♀	Gene	2149.69	7	307.10	497.34	0.000000
		Concentration	23.68	4	5.92	9.59	0.000000
		Gene × Concentration	31.29	28	1.12	1.81	0.008564
		Error	196.36	318	0.62		
ML7	♀	Gene	1227.88	7	175.41	203.32	0.000000
		Concentration	8.95	3	2.98	3.46	0.017056
		Gene × Concentration	20.45	21	0.97	1.13	0.317857
		Error	220.85	256	0.86		
CD	♀	Gene	1127.89	7	161.13	267.66	0.000000
		Concentration	5.01	2	2.51	4.16	0.016996
		Gene × Concentration	9.50	14	0.68	1.13	0.336278
		Error	113.17	188	0.60		
AI	♀	Gene	1029.68	7	147.10	238.92	0.000000
		Concentration	6.97	2	3.49	5.66	0.004083
		Gene × Concentration	4.54	14	0.32	0.53	0.915857
		Error	117.59	191	0.62		

VP – Verteporfin; ML7 – ML-7 hydrochloride; CD – Cytochalasin D; AI – AICAR; ♂ – male; ♀ – female; SS – sum-of-squares; DF – degrees of freedom; MS – mean squares. Results of F-tests: F – F-value and p – p-value.
