# Supplementary material for: Studying the Geroprotective Properties of YAP/TAZ Signaling Inhibitors on Drosophila melanogaster Model
Source: Int J Mol Sci. 2023 Mar 22;24(6):6006. doi: 10.3390/ijms24066006 (PMC10058302; doi:10.3390/ijms24066006)
Supplement: Supplementary file 1 [file ijms-24-06006-s001.zip › Table S7.pdf]

Table S7. The expression level of Yki target genes in 20-day-old males after treatment with YAP/TAZ inhibitors.

Gene	Concentration (μM)	Mean (dCt)	SE	DT (p)
Verteporfin				
CycE	0 (control)	12.54	0.27	
	0.01	12.59	0.15	0.8868
	0.1	12.11	0.21	0.2748
	1	12.48	0.17	0.8704
	10	12.30	0.14	0.5192
dally	0 (control)	7.32	0.19	
	0.01	7.29	0.32	0.9264
	0.1	7.26	0.11	0.8628
	1	7.77	0.23	0.2844
	10	7.41	0.15	0.7959
Diap1	0 (control)	3.57	0.16	
	0.01	3.10	0.14	0.1855
	0.1	3.81	0.21	0.5553
	1	3.78	0.06	0.5928
	10	3.73	0.09	0.6390
E2f1	0 (control)	4.09	0.22	
	0.01	3.73	0.21	0.3550
	0.1	4.60	0.24	0.2203
	1	4.57	0.19	0.2384
	10	4.52	0.12	0.2530
myc	0 (control)	6.99	0.15	
	0.01	6.27	0.04	0.0670
	0.1	6.27	0.16	0.0602
	1	6.57	0.07	0.2622
	10	6.65	0.16	0.3374
vn	0 (control)	7.84	0.14	
	0.01	7.76	0.18	0.8368
	0.1	7.42	0.13	0.3111
	1	7.76	0.09	0.8384
	10	7.77	0.06	0.8513
wg	0 (control)	8.14	0.59	
	0.01	9.95	0.65	0.0000↓
	0.1	8.60	0.75	0.1955
	1	7.68	0.25	0.2634
	10	8.71	0.23	0.1294
Yki	0 (control)	4.81	0.25	
	0.01	4.56	0.17	0.5303
	0.1	4.48	0.05	0.4309
	1	4.86	0.11	0.8881
	10	5.03	0.16	0.5640
ML-7				
CycE	0 (control)	13.38	0.35	
	0.01	13.81	0.41	0.4932
	0.1	13.13	0.23	0.6885
	1	11.35	0.87	0.0017↑
dally	0 (control)	8.06	0.13	
	0.01	8.25	0.20	0.7870
	0.1	7.60	0.11	0.5004
	1	8.11	0.14	0.9355
Diap1	0 (control)	3.63	0.28	
	0.01	2.77	0.28	0.1991
	0.1	3.14	0.13	0.4395

	1	3.80	0.23	0.7852
E2f1	0 (control)	3.98	0.10	
	0.01	3.87	0.25	0.8587
	0.1	4.20	0.19	0.7280
	1	4.23	0.11	0.7182
myc	0 (control)	6.93	0.10	
	0.01	7.18	0.28	0.7275
	0.1	7.10	0.27	0.7981
	1	7.06	0.19	0.8326
vn	0 (control)	8.29	0.33	
	0.01	8.07	0.31	0.7490
	0.1	8.70	0.28	0.5160
	1	4.45	1.72	0.0000↑
wg	0 (control)	7.52	0.14	
	0.01	6.62	0.78	0.2188
	0.1	7.62	0.48	0.8808
	1	9.44	0.37	0.0074↓
Yki	0 (control)	5.21	0.15	
	0.01	4.85	0.18	0.5935
	0.1	4.43	0.17	0.2759
	1	5.02	0.24	0.7625
Cytochalasin D				
CycE	0 (control)	12.49	0.32	
	0.1	12.96	0.39	0.3198
	1	13.00	0.33	0.3108
dally	0 (control)	7.96	0.30	
	0.1	6.79	0.28	0.0292
	1	7.67	0.18	0.5632
Diap1	0 (control)	3.14	0.16	
	0.1	2.78	0.12	0.4505
	1	3.39	0.33	0.6203
E2f1	0 (control)	3.69	0.32	
	0.1	3.19	0.25	0.3358
	1	3.40	0.30	0.5338
myc	0 (control)	6.43	0.25	
	0.1	5.41	0.34	0.0387
	1	6.74	0.34	0.5083
vn	0 (control)	7.47	0.27	
	0.1	7.80	0.29	0.5360
	1	7.43	0.22	0.9299
wg	0 (control)	7.50	0.65	
	0.1	5.95	0.66	0.0028↑
	1	8.01	0.43	0.3513
Yki	0 (control)	4.56	0.22	
	0.1	4.68	0.23	0.7915
	1	4.38	0.19	0.7049
AICAR				
CycE	0 (control)	12.56	0.13	
	0.1	12.84	0.19	0.4536
	1	12.06	0.11	0.1704
dally	0 (control)	7.82	0.15	
	0.1	7.96	0.14	0.7353
	1	7.85	0.38	0.9350
Diap1	0 (control)	3.49	0.30	
	0.1	3.59	0.26	0.7687
	1	3.61	0.23	0.7572
E2f1	0 (control)	4.09	0.09	

	0.1	4.28	0.19	0.6146
	1	4.63	0.30	0.1844
myc	0 (control)	6.93	0.33	
	0.1	6.54	0.16	0.3142
	1	6.64	0.18	0.4254
vn	0 (control)	7.95	0.10	
	0.1	8.09	0.10	0.7231
	1	8.01	0.11	0.8646
wg	0 (control)	8.51	0.51	
	0.1	8.58	0.46	0.8534
	1	8.73	0.42	0.5803
Yki	0 (control)	4.77	0.26	
	0.1	4.83	0.29	0.8725
	1	4.50	0.18	0.4865

dCt – delta Ct (cycle thresholds) values were calculated as the differences in Ct values for target genes and reference genes (*β-Tubulin* and *RpL32*). The Ct values are inversely proportional to the mRNA transcript levels. Higher dCt values represent a lower expression level. SE – standard error of mean, p – p-value. Significance was determined by two-way ANOVA followed by post-hoc Duncan test (DT), ↑ – increase, ↓ – decrease.
