# Supplementary material for: Studying the Geroprotective Properties of YAP/TAZ Signaling Inhibitors on Drosophila melanogaster Model
Source: Int J Mol Sci. 2023 Mar 22;24(6):6006. doi: 10.3390/ijms24066006 (PMC10058302; doi:10.3390/ijms24066006)
Supplement: Supplementary file 1 [file ijms-24-06006-s001.zip › Table S8.pdf]

Table S8. The expression level of Yki target genes in 20-day-old females after treatment with YAP/TAZ inhibitors.

Gene	Concentration (μM)	Mean (dCt)	SE	DT (p)
Verteporfin				
CycE	0 (control)	10.71	0.14	
	0.01	10.79	0.23	0.8265
	0.1	11.31	0.32	0.1528
	1	11.59	0.19	0.0366↓
	10	11.20	0.20	0.2333
dally	0 (control)	8.93	0.11	
	0.01	9.48	0.14	0.2007
	0.1	9.79	0.36	0.0504↓
	1	10.10	0.21	0.0061↓
	10	9.56	0.16	0.1490
Diap1	0 (control)	4.59	0.21	
	0.01	4.24	0.06	0.4294
	0.1	4.53	0.38	0.8825
	1	5.04	0.25	0.3117
	10	4.66	0.11	0.8599
E2f1	0 (control)	4.57	0.19	
	0.01	5.06	0.21	0.2824
	0.1	4.34	0.37	0.5712
	1	5.02	0.26	0.3117
	10	4.89	0.08	0.4705
myc	0 (control)	6.89	0.20	
	0.01	6.86	0.10	0.9483
	0.1	6.07	0.12	0.0376
	1	7.00	0.12	0.7618
	10	7.11	0.24	0.5793
vn	0 (control)	8.72	0.10	
	0.01	8.78	0.12	0.8887
	0.1	8.77	0.12	0.9035
	1	9.35	0.21	0.1483
	10	9.09	0.09	0.3886
wg	0 (control)	9.58	0.41	
	0.01	9.27	0.53	0.4832
	0.1	9.43	0.74	0.7226
	1	10.99	0.69	0.0004↓
	10	8.70	0.18	0.0495↑
Yki	0 (control)	4.66	0.14	
	0.01	4.78	0.07	0.7383
	0.1	4.24	0.16	0.3512
	1	4.99	0.10	0.4294
	10	4.64	0.15	0.9677
ML-7				
CycE	0 (control)	10.56	0.36	
	0.01	9.60	0.33	0.0536
	0.1	10.46	0.42	0.8189
	1	10.11	0.27	0.3355
dally	0 (control)	8.48	0.19	
	0.01	8.35	0.31	0.7897
	0.1	8.61	0.28	0.7623
	1	9.19	0.41	0.1413
Diap1	0 (control)	4.66	0.20	
	0.01	3.98	0.30	0.1415

	0.1	4.16	0.05	0.2516
	1	4.68	0.16	0.9701
E2f1	0 (control)	5.37	0.14	
	0.01	5.12	0.16	0.6055
	0.1	4.91	0.06	0.3794
	1	5.20	0.17	0.7243
myc	0 (control)	6.39	0.15	
	0.01	6.16	0.44	0.6179
	0.1	6.39	0.09	0.9943
	1	7.08	0.23	0.1140
vn	0 (control)	9.83	0.08	
	0.01	9.78	0.27	0.9103
	0.1	9.11	0.18	0.1554
	1	9.47	0.21	0.4609
wg	0 (control)	7.26	0.50	
	0.01	7.39	0.83	0.7567
	0.1	8.05	0.56	0.0847
	1	8.37	0.40	0.0208↓
Yki	0 (control)	5.36	0.19	
	0.01	5.04	0.24	0.5258
	0.1	4.83	0.06	0.3064
	1	5.05	0.11	0.5293
Cytochalasin D				
CycE	0 (control)	10.50	0.15	
	0.1	11.66	0.28	0.0024↓
	1	10.70	0.16	0.5953
dally	0 (control)	9.32	0.19	
	0.1	9.57	0.11	0.5406
	1	9.61	0.32	0.4958
Diap1	0 (control)	4.89	0.26	
	0.1	4.89	0.13	0.9963
	1	4.64	0.16	0.4962
E2f1	0 (control)	5.11	0.23	
	0.1	5.03	0.29	0.8377
	1	5.30	0.26	0.6415
myc	0 (control)	6.70	0.12	
	0.1	6.86	0.13	0.6683
	1	7.02	0.22	0.4288
vn	0 (control)	9.07	0.15	
	0.1	9.63	0.24	0.2045
	1	9.37	0.21	0.4746
wg	0 (control)	9.40	0.63	
	0.1	10.20	0.39	0.0499↓
	1	9.25	0.42	0.7288
Yki	0 (control)	5.11	0.12	
	0.1	5.21	0.17	0.7917
	1	5.11	0.20	0.9873
AICAR				
CycE	0 (control)	9.58	0.23	
	0.1	8.99	0.16	0.1699
	1	9.44	0.17	0.7290
dally	0 (control)	8.65	0.10	
	0.1	8.45	0.12	0.5807
	1	8.18	0.49	0.2364
Diap1	0 (control)	4.60	0.04	
	0.1	4.29	0.03	0.4443
	1	4.34	0.05	0.4829

E2f1	0 (control)	5.23	0.05	
	0.1	4.82	0.07	0.3406
	1	4.81	0.16	0.3341
myc	0 (control)	6.54	0.08	
	0.1	5.99	0.12	0.1573
	1	6.38	0.36	0.6680
vn	0 (control)	9.26	0.04	
	0.1	9.10	0.21	0.6763
	1	9.52	0.23	0.5079
wg	0 (control)	10.80	0.14	
	0.1	9.87	0.51	0.0158↑
	1	10.09	0.82	0.0544
Yki	0 (control)	5.21	0.06	
	0.1	4.90	0.07	0.3926
	1	4.84	0.14	0.3439

dCt – delta Ct (cycle thresholds) values were calculated as the differences in Ct values for target genes and reference genes (*β-Tubulin* and *RpL32*). The Ct values are inversely proportional to the mRNA transcript levels. Higher dCt values represent a lower expression level. SE – standard error of mean, p – p-value. Significance was determined by two-way ANOVA followed by post-hoc Duncan test (DT).
