# Supplementary material for: Studying the Geroprotective Properties of YAP/TAZ Signaling Inhibitors on Drosophila melanogaster Model
Source: Int J Mol Sci. 2023 Mar 22;24(6):6006. doi: 10.3390/ijms24066006 (PMC10058302; doi:10.3390/ijms24066006)
Supplement: Supplementary file 1 [file ijms-24-06006-s001.zip › Table S9.pdf]

Table S9. Effects of treatment with solvents on the lifespan of control groups of male and female flies. The results of two independent replicate experiments are presented.

Solvent (v/v)	Sex	M (days)	dM (%)	FET (M)		90% (days)	d90% (%)	FET (90%)		FHT (Early)		FHT (Late)		N
				p	pB			p	pB	p	pB	p	pB	
100% water	♂	36	0	n/a	n/a	40	n/a	n/a	n/a	n/a	n/a	n/a	n/a	293
100% ethanol	♂	36	0	1	1	40	0	0.71	1	0.98	1	0.719	1	278
50% ethanol	♂	35	-3	0.23	0.69	40	0	0.02	0.06	0.0038	0.015	0.028	0.08	290
10% Cyrene	♂	36	0	0.0023	0.0069	41	3	0.0003	0.0009	0.0372	0.1117	0.0935	1	287
100% water	♀	35	0	n/a	n/a	40	n/a	n/a	n/a	n/a	n/a	n/a	n/a	323
100% ethanol	♀	35	0	0.014	0.042	40	0	0	0	0.003	0.009	0	0	292
50% ethanol	♀	35	0	0.56	1	40	0	0.0001	0.0004	1	1	0.023	0.0689	300
10% Cyrene	♀	36	3	0	0	43	8	0	0	0.0004	0.0011	0	0	282

Solvent (v/v) – volume concentration of solvent; ♂ – male; ♀ – female; M (days) – median lifespan; 90% (days) – maximum lifespan (age of 90% mortality); dM, d90% – differences between median and maximum lifespan of the control and experimental flies, respectively; p – p-value; pB – Bonferroni-corrected p-value; FET (M) and FET (90%) – Fisher's Exact Test for median and maximum lifespan, respectively; FHT (Early) and FHT (Late) – Fleming-Harrington Test sensitive against early and later differences, respectively; n/a – not applicable; N – number of flies.
