# Supplementary material for: Studying the Geroprotective Properties of YAP/TAZ Signaling Inhibitors on Drosophila melanogaster Model
Source: Int J Mol Sci. 2023 Mar 22;24(6):6006. doi: 10.3390/ijms24066006 (PMC10058302; doi:10.3390/ijms24066006)
Supplement: Supplementary file 1 [file ijms-24-06006-s001.zip › Table S10.pdf]

Table S10. Effects of treatment with YAP/TAZ inhibitors on the lifespan of male and female flies. The results of two independent replicate experiments are presented.

Inhibitor	Sex	Rep.	C (μM)	M (days)	dM (%)	FET (M)		90% (days)	d90% (%)	FET (90%)		FHT (Early)		FHT (Late)		N
						p	pB			p	pB	p	pB	p	pB	
VP	♂	1	0 (control)	36	n/a	n/a	n/a	37	n/a	n/a	n/a	n/a	n/a	n/a	n/a	145
			0.01	36	0	0.2	0.4	38	+3	1	1	0.5216	1	0.2043	0.4086	141
			0.1	36	0	0.002	0.004	40	+8	0	0	0.0009	0.0019	0	0	154
VP	♂	2	0 (control)	36	n/a	n/a	n/a	41	n/a	n/a	n/a	n/a	n/a	n/a	n/a	152
			0.01	37	+3	0.08	0.16	42	+2	1	1	0.0024	0.0048	0	0	165
			0.1	37	+3	0.05	0.1	42	+2	0	0	0.011	0.0221	0	0	159
ML	♂	1	0 (control)	35	n/a	n/a	n/a	40	n/a	n/a	n/a	n/a	n/a	n/a	n/a	147
			0.1	35	0	0.9	1	40	0	0.7	1	0.53	1	0.0722	0.1445	144
			1	36	+3	0.7	1	40	0	0.2	1	0.667	1	0.1454	0.2909	153
ML	♂	2	0 (control)	35	n/a	n/a	n/a	40	n/a	n/a	n/a	n/a	n/a	n/a	n/a	153
			0.1	36	+3	0.3	0.6	41	+3	0	0	0.132	0.264	0.0135	0.027	143
			1	36	+3	0.4	0.8	37	-7	1	1	0.597	1	0.0021	0.0041	156
CD	♂	1	0 (control)	36	n/a	n/a	n/a	40	n/a	n/a	n/a	n/a	n/a	n/a	n/a	140
			0.1	37	+3	0.003	0.006	41	+3	0.01	0.02	0.0003	0.0006	0.0003	0.0006	163
			1	37	+3	0.022	0.044	41	+3	0.015	0.03	0.0643	0.1286	0.0643	0.1286	153
CD	♂	2	0 (control)	36	n/a	n/a	n/a	40	n/a	n/a	n/a	n/a	n/a	n/a	n/a	150
			0.1	37	+3	0.34	0.68	41	+3	0.6	1	0.038	0.076	0.1021	0.2042	161
			1	37	+3	0.47	0.54	41	+3	1	1	0.005	0.01	0.0003	0.0005	150
AI	♂	1	0 (control)	35	n/a	n/a	n/a	40	n/a	n/a	n/a	n/a	n/a	n/a	n/a	150
			0.1	37	+6	0.015	0.03	41	+3	0	0	0	0	0	0	174
			1	37	+6	0.0002	0.0004	40	0	0.0002	0.0004	0.0008	0.0016	0.0013	0.0025	157
AI	♂	2	0 (control)	40	n/a	n/a	n/a	40	n/a	n/a	n/a	n/a	n/a	n/a	n/a	143
			0.1	37	-7	0.02	0.04	41	+3	0.04	0.08	0.062	0.12	0.0037	0.0073	147
			1	37	-7	0.005	0.01	41	+3	0.01	0.02	0.041	0.08	0.0001	0.0001	156
VP	♀	1	0 (control)	36	n/a	n/a	n/a	39	n/a	n/a	n/a	n/a	n/a	n/a	n/a	147
			0.01	36	0	0.09	0.18	40	+3	0.12	0.24	0.0354	0.0708	0.4701	0.9402	157
			0.1	36	0	0.14	0.24	40	+3	0.79	1	0.1807	0.3613	0.1031	0.2062	150
VP	♀	2	0 (control)	36	n/a	n/a	n/a	41	n/a	n/a	n/a	n/a	n/a	n/a	n/a	145
			0.01	34	-6	0.02	0.04	41	0	0.001	0.002	0.151	0.3021	0.7784	1	148
			0.1	36	0	1	1	42	+3	0	0	0.6765	1	0.0079	0.0158	131
ML	♀	1	0 (control)	35	n/a	n/a	n/a	40	n/a	n/a	n/a	n/a	n/a	n/a	n/a	142
			0.1	35	0	0.5	1	41	+3	0.2	1	0.812	1	0.0095	0.0189	136

			1	35	0	0.4	1	40	0	0.4	1	0.803	1	0.574	1	140
ML	♀	2	0 (control)	34	n/a	n/a	n/a	40	n/a	n/a	n/a	n/a	n/a	n/a	n/a	158
			0.1	34	0	0.12	0.24	37	-7	0.004	0.008	0.17	0.34	0.809	1	138
			1	35	+3	0.29	0.58	40	0	0	0	0.0704	0.14	0.0064	0.0129	143
CD	♀	1	0 (control)	34	n/a	n/a	n/a	40	n/a	n/a	n/a	n/a	n/a	n/a	n/a	149
			0.1	33	-3	0.9	1	40	0	0.6	1	0.102	0.205	0.3496	0.6992	152
			1	33	-3	0.013	0.026	40	0	0.001	0.002	0.0016	0.0032	0.0121	0.0243	168
CD	♀	2	0 (control)	35	n/a	n/a	n/a	40	n/a	n/a	n/a	n/a	n/a	n/a	n/a	143
			0.1	34	-3	0.006	0.012	40	0	0.007	0.014	0.016	0.032	0.0004	0.0007	141
			1	36	+3	0.02	0.04	41	+3	0.04	0.08	0.045	0.096	0.2886	0.5772	162
AI	♀	1	0 (control)	34	n/a	n/a	n/a	37	n/a	n/a	n/a	n/a	n/a	n/a	n/a	163
			0.1	34	0	0.005	0.01	37	0	0.35	1	0.089	0.171	0.3784	0.7569	146
			1	37	+9	01	02	40	+8	1	1	0	0	0	0	161
AI	♀	2	0 (control)	35	n/a	n/a	n/a	40	n/a	n/a	n/a	n/a	n/a	n/a	n/a	160
			0.1	34	-3	0.431	0.862	37	-8	1	1	0.28	0.567	0.0824	0.1647	164
			1	37	+6	0.0001	0.0002	40	0	0.0009	0.0018	0.0033	0.0067	0.001	0.0021	169

VP – Verteporfin; ML7 – ML-7 hydrochloride; CD – Cytochalasin D; AI – AICAR; ♂ – male; ♀ – female; Rep. – Replication; C (μM) – Concentration (μM); M (days) – median lifespan; 90% (days) – maximum lifespan (age of 90% mortality); dM, d90% – differences between median and maximum lifespan of the control and experimental flies, respectively; p – p-value; pB – Bonferroni-corrected p-value; FET (M) and FET (90%) – Fisher's Exact Test for median and maximum lifespan, respectively; FHT (Early) and FHT (Late) – Fleming-Harrington test sensitive against early and later differences, respectively; n/a – not applicable; N – number of flies.
