# Supplementary material for: Studying the Geroprotective Properties of YAP/TAZ Signaling Inhibitors on Drosophila melanogaster Model
Source: Int J Mol Sci. 2023 Mar 22;24(6):6006. doi: 10.3390/ijms24066006 (PMC10058302; doi:10.3390/ijms24066006)
Supplement: Supplementary file 1 [file ijms-24-06006-s001.zip › Table S11.pdf]

Table S11. Effects of treatment with YAP/TAZ inhibitors on the lifespan of male and female flies. The results from 2 independent experiments are pooled.

Inhibitor	Sex	C (μM)	M (days)	dM (%)	FET (M)		90% (days)	d90% (%)	FET (90%)		FHT (Early)		FHT (Late)		N
					p	pB			p	pB	p	pB	p	pB	
VP	♂	0 (control)	36	n/a	n/a	n/a	41	n/a	n/a	n/a	n/a	n/a	n/a	n/a	287
		0.01	36	0	0.007	0.014	42	+2	0	0	0.0079	0.0158	0.0002	0.0005	306
		0.1	36	0	0.007	0.014	42	+2	0	0	0.0002	0.0004	0	0	293
ML	♂	0 (control)	35	n/a	n/a	n/a	40	n/a	n/a	n/a	n/a	n/a	n/a	n/a	290
		0.1	36	+3	0.6	1	40	0	0	0.0002	0.503	1	0.444	0.888	277
		1	36	+3	0.4	0.8	40	0	0.2	0.4	0.551	1	0.0015	0.003	299
CD	♂	0 (control)	36	n/a	n/a	n/a	40	n/a	n/a	n/a	n/a	n/a	n/a	n/a	278
		0.1	37	+3	0.006	0.012	41	+3	0.042	0.084	0.0001	0.0001	0.0001	0.0003	324
		1	37	+3	0.029	0.058	41	+3	0.001	0.002	0.0012	0.0023	0.0002	0.0004	303
AI	♂	0 (control)	36	n/a	n/a	n/a	40	n/a	n/a	n/a	n/a	n/a	n/a	n/a	293
		0.1	37	+3	0.2	0.4	41	+3	0.01	0.02	0.0004	0.0007	0	0	321
		1	37	+3	0.4	0.8	41	+3	0.03	0.06	0.0007	0.0015	0	0	293
VP	♀	0 (control)	36	n/a	n/a	n/a	41	n/a	n/a	n/a	n/a	n/a	n/a	n/a	282
		0.01	36	0	0.6	1	40	-3	0.3	0.6	0.9501	1	0.0832	0.1665	305
		0.1	36	0	0.2	1	41	0	0.0008	0.0016	0.3839	0.7679	0.1785	0.357	281
ML	♀	0 (control)	35	n/a	n/a	n/a	40	n/a	n/a	n/a	n/a	n/a	n/a	n/a	300
		0.1	34	+3	0.2	0.4	40	0	0.009	0.018	0.39	0.789	0.0212	0.0424	274
		1	35	0	0.1	0.2	40	0	0.05	0.1	0.15	0.3	0.0167	0.0335	283
CD	♀	0 (control)	35	n/a	n/a	n/a	40	n/a	n/a	n/a	n/a	n/a	n/a	n/a	292
		0.1	34	-3	0.04	0.08	40	0	0.34	0.64	0.004	0.007	0.2285	0.4569	293
		1	35	0	0.8	1	40	0	1	1	0.46	0.92	0.5862	1	330
AI	♀	0 (control)	35	n/a	n/a	n/a	40	n/a	n/a	n/a	n/a	n/a	n/a	n/a	323
		0.1	34	+3	0.001	0.002	37	-8	1	1	0.087	0.17	0.0999	0.1998	310
		1	37	+6	0	0	40	0	0.0001	0.0002	0	0	0	0	330

VP – Verteporfin; ML7 – ML-7 hydrochloride; CD – Cytochalasin D; AI – AICAR; ♂ – male; ♀ – female; Rep. – Replication; C (μM) – Concentration (μM); M (days) – median lifespan; 90% (days) – maximum lifespan (age of 90% mortality); dM, d90% – differences between median and maximum lifespan of the control and experimental flies, respectively; p – p-value; pB – Bonferroni-corrected p-value; FET (M) and FET (90%) – Fisher's Exact Test for median and maximum lifespan, respectively; FHT (Early) and FHT (Late) – Fleming-Harrington test sensitive against early and later differences in deaths, respectively; n/a – not applicable; N – number of flies.
