# Supplementary material for: Studying the Geroprotective Properties of YAP/TAZ Signaling Inhibitors on Drosophila melanogaster Model
Source: Int J Mol Sci. 2023 Mar 22;24(6):6006. doi: 10.3390/ijms24066006 (PMC10058302; doi:10.3390/ijms24066006)
Supplement: Supplementary file 1 [file ijms-24-06006-s001.zip › Table S12.pdf]

**Table S12.** Tree-way ANOVA of the effect of Yap/Taz inhibitors on the expression level of antimicrobial genes *AttA*, *DptA*, *CecA1* and Wnt-related gene *dpp*.

| Substance | Sex | Source of variation | SS | DF | MS | F | p |
|---|---|---|---|---|---|---|---|
| VP | ♂ | Gene | 1291.149 | 3 | 430.383 | 716.05 | 0.0000 |
| | | Concentration | 5.029 | 2 | 2.515 | 4.18 | 0.0167 |
| | | Age | 24.789 | 1 | 24.789 | 41.24 | 0.0000 |
| | | Gene × Concentration | 13.927 | 6 | 2.321 | 3.86 | 0.0012 |
| | | Gene × Age | 54.670 | 3 | 18.223 | 30.32 | 0.0000 |
| | | Concentration × Age | 8.950 | 2 | 4.475 | 7.45 | 0.0008 |
| | | Gene × Concentration × Age | 7.031 | 6 | 1.172 | 1.95 | 0.0749 |
| | | Error | 113.599 | 189 | 0.601 | | |
| ML7 | ♂ | Gene | 998.631 | 3 | 332.877 | 102.524 | 0.0000 |
| | | Concentration | 25.997 | 2 | 12.998 | 4.003 | 0.0198 |
| | | Age | 28.596 | 1 | 28.596 | 8.807 | 0.0034 |
| | | Gene × Concentration | 15.396 | 6 | 2.566 | 0.790 | 0.5786 |
| | | Gene × Age | 66.178 | 3 | 22.059 | 6.794 | 0.0002 |
| | | Concentration × Age | 10.629 | 2 | 5.315 | 1.637 | 0.1974 |
| | | Gene × Concentration × Age | 22.044 | 6 | 3.674 | 1.132 | 0.3456 |
| | | Error | 603.911 | 186 | 3.247 | | |
| CD | ♂ | Gene | 2378.750 | 3 | 792.917 | 360.705 | 0.0000 |
| | | Concentration | 31.259 | 2 | 15.629 | 7.110 | 0.0010 |
| | | Age | 141.140 | 1 | 141.140 | 64.206 | 0.0000 |
| | | Gene × Concentration | 81.183 | 6 | 13.530 | 6.155 | 0.0000 |
| | | Gene × Age | 142.516 | 3 | 47.505 | 21.611 | 0.0000 |
| | | Concentration × Age | 17.526 | 2 | 8.763 | 3.986 | 0.0201 |
| | | Gene × Concentration × Age | 15.475 | 6 | 2.579 | 1.173 | 0.3222 |
| | | Error | 422.062 | 192 | 2.198 | | |
| AI | ♂ | Gene | 3646.858 | 3 | 1215.619 | 384.355 | 0.0000 |
| | | Concentration | 0.415 | 2 | 0.208 | 0.066 | 0.9365 |
| | | Age | 90.876 | 1 | 90.876 | 28.733 | 0.0000 |
| | | Gene × Concentration | 18.633 | 6 | 3.105 | 0.982 | 0.4388 |
| | | Gene × Age | 116.488 | 3 | 38.829 | 12.277 | 0.0000 |
| | | Concentration × Age | 66.143 | 2 | 33.072 | 10.457 | 0.0000 |
| | | Gene × Concentration × Age | 27.352 | 6 | 4.559 | 1.441 | 0.2008 |
| | | Error | 597.761 | 189 | 3.163 | | |
| VP | ♀ | Gene | 42.53 | 3 | 14.18 | 31.40 | 0.0000 |
| | | Concentration | 3.72 | 2 | 1.86 | 4.12 | 0.0176 |
| | | Age | 352.97 | 1 | 352.97 | 781.86 | 0.0000 |
| | | Gene × Concentration | 21.60 | 6 | 3.60 | 7.97 | 0.0000 |
| | | Gene × Age | 46.96 | 3 | 15.65 | 34.68 | 0.0000 |
| | | Concentration × Age | 4.95 | 2 | 2.48 | 5.49 | 0.0048 |
| | | Gene × Concentration × Age | 3.04 | 6 | 0.51 | 1.12 | 0.3501 |
| | | Error | 85.32 | 189 | 0.45 | | |
| ML7 | ♀ | Gene | 322.204 | 3 | 107.401 | 324.95 | 0.0000 |
| | | Concentration | 15.619 | 2 | 7.809 | 23.63 | 0.0000 |
| | | Age | 39.907 | 1 | 39.907 | 120.74 | 0.0000 |
| | | Gene × Concentration | 4.940 | 6 | 0.823 | 2.49 | 0.0242 |
| | | Gene × Age | 3.454 | 3 | 1.151 | 3.48 | 0.0170 |
| | | Concentration × Age | 17.291 | 2 | 8.645 | 26.16 | 0.0000 |
| | | Gene × Concentration × Age | 5.955 | 6 | 0.993 | 3.00 | 0.0080 |
| | | Error | 62.137 | 188 | 0.331 | | |
| CD | ♀ | Gene | 11.73 | 3 | 3.91 | 3.28 | 0.0221 |
| | | Concentration | 4.10 | 2 | 2.05 | 1.72 | 0.1820 |
| | | Age | 198.98 | 1 | 198.98 | 166.83 | 0.0000 |
| | | Gene × Concentration | 31.12 | 6 | 5.19 | 4.35 | 0.0004 |
| | | Gene × Age | 162.44 | 3 | 54.15 | 45.40 | 0.0000 |
| | | Concentration × Age | 17.51 | 2 | 8.75 | 7.34 | 0.0008 |

| | | | | | | | |
|---|---|---|---|---|---|---|---|
| | | Gene × Concentration × Age | 42.87 | 6 | 7.14 | 5.99 | 0.0000 |
| | | Error | 229.00 | 192 | 1.19 | | |
| AI | ♀ | Gene | 711.50 | 3 | 237.17 | 218.81 | 0.0000 |
| | | Concentration | 28.23 | 2 | 14.11 | 13.02 | 0.0000 |
| | | Age | 235.89 | 1 | 235.89 | 217.64 | 0.0000 |
| | | Gene × Concentration | 35.99 | 6 | 6.00 | 5.53 | 0.0000 |
| | | Gene × Age | 194.96 | 3 | 64.99 | 59.96 | 0.0000 |
| | | Concentration × Age | 22.82 | 2 | 11.41 | 10.53 | 0.0000 |
| | | Gene × Concentration × Age | 14.84 | 6 | 2.47 | 2.28 | 0.0376 |
| | | Error | 207.02 | 191 | 1.08 | | |

VP – Verteporfin; ML7 – ML-7 hydrochloride; CD – Cytochalasin D; AI – AICAR; ♂ – male; ♀ – female; SS – sum-of-squares; DF – degrees of freedom; MS – mean squares. Results of F-tests: F – F-value and p – p-value.
