# Supplementary material for: Studying the Geroprotective Properties of YAP/TAZ Signaling Inhibitors on Drosophila melanogaster Model
Source: Int J Mol Sci. 2023 Mar 22;24(6):6006. doi: 10.3390/ijms24066006 (PMC10058302; doi:10.3390/ijms24066006)
Supplement: Supplementary file 1 [file ijms-24-06006-s001.zip › Table S13.pdf]

Table S13. Effect of Yap/Taz inhibitors on the expression level of antimicrobial genes *AttA*, *DptA*, *CecA1* and Wnt-related gene *dpp* in *Drosophila* males.

Gene	C (µM)	Age (days)	Mean (dCt)	SE	DT (p)	
					AE	TE
Verteporfin						
Atta	control	10	6.87	0.46	n/a	n/a
	0.01		6.74	0.54	n/a	0.7417
	0.1		7.75	0.13	n/a	0.0453 ↓
	control	20	5.39	0.36	0.0002 ↑	n/a
	0.01		6.91	0.19	0.6847	0.0002 ↓
	0.1		5.92	0.25	0.0000 ↑	0.1751
DptA	control	10	7.04	0.46	n/a	n/a
	0.01		7.46	0.43	n/a	0.3290
	0.1		8.41	0.18	n/a	0.0008 ↓
	control	20	4.86	0.15	0.0000 ↑	n/a
	0.01		6.14	0.09	0.0016 ↑	0.0012 ↓
	0.1		5.70	0.27	0.0000 ↑	0.0310 ↓
CecA1	control	10	0.94	0.08	n/a	n/a
	0.01		0.64	0.18	n/a	0.4268
	0.1		0.36	0.11	n/a	0.1393
	control	20	1.54	0.13	0.1339	n/a
	0.01		1.32	0.09	0.0917	0.5505
	0.1		1.32	0.12	0.0150 ↓	0.5729
dpp	control	10	7.46	0.11	n/a	n/a
	0.01		7.35	0.36	n/a	0.7759
	0.1		7.44	0.16	n/a	0.9474
	control	20	7.03	0.17	0.3218	n/a
	0.01		7.34	0.12	0.9911	0.4319
	0.1		6.74	0.06	0.1119	0.4806
ML-7						
Atta	control	10	5.18	0.47	n/a	n/a
	0.1		5.08	0.26	n/a	0.9145
	1		5.71	0.34	n/a	0.5601
	control	20	3.79	0.40	0.1984	n/a
	0.1		2.75	0.38	0.0303 ↑	0.3192
	1		3.45	0.17	0.0348 ↑	0.7118
DptA	control	10	4.99	0.26	n/a	n/a
	0.1		3.94	0.27	n/a	0.2830
	1		5.06	0.73	n/a	0.9374
	control	20	3.30	0.29	0.1085	n/a
	0.1		3.05	0.49	0.3955	0.7824
	1		4.25	0.10	0.4041	0.3688
CecA1	control	10	0.85	0.11	n/a	n/a
	0.1		0.88	0.08	n/a	0.9790
	1		2.08	0.09	n/a	0.2087
	control	20	0.28	0.08	0.5564	n/a
	0.1		0.40	0.10	0.6259	0.8934
	1		0.10	0.15	0.0576	0.8486
dpp	control	10	7.33	0.33	n/a	n/a
	0.1		6.25	0.86	n/a	0.2393
	1		8.23	0.38	n/a	0.3746
	control	20	9.85	2.13	0.0120 ↓	n/a

	0.1		7.34	0.11	0.2626	0.0108 ↑
	1		7.63	0.31	0.5080	0.0207 ↑
Cytohalasin D						
Atta	control	10	9.67	0.13	n/a	n/a
	0.1		8.31	0.45	n/a	0.0820
	1		8.13	0.22	n/a	0.0524
	control	20	6.70	0.71	0.0001 ↑	n/a
	0.1		5.26	0.74	0.0000 ↑	0.0499 ↑
	1		5.75	0.54	0.0012 ↑	0.1754
DptA	control	10	9.56	0.10	n/a	n/a
	0.1		8.64	0.41	n/a	0.2183
	1		9.29	0.19	n/a	0.6990
	control	20	7.23	0.50	0.0023 ↑	n/a
	0.1		4.34	0.41	0.0000 ↑	0.0001 ↑
	1		5.18	0.37	0.0000 ↑	0.0068 ↑
CecA1	control	10	0.94	0.10	n/a	n/a
	0.1		3.65	1.40	n/a	0.0003
	1		0.87	0.07	n/a	0.9162
	control	20	1.07	0.20	0.8602	n/a
	0.1		1.88	0.15	0.0114 ↑	0.2759
	1		1.11	0.11	0.7620	0.9536
dpp	control	10	10.46	0.20	n/a	n/a
	0.1		11.34	0.67	n/a	0.2385
	1		10.14	0.18	n/a	0.6503
	control	20	12.03	0.53	0.0388 ↓	n/a
	0.1		11.27	0.79	0.9239	0.3109
	1		9.78	0.11	0.6084	0.0034 ↑
AICAR						
Atta	control	10	7.22	0.48	n/a	n/a
	0.1		8.33	0.30	n/a	0.2347
	1		7.19	0.38	n/a	0.9728
	control	20	5.62	0.52	0.0833	n/a
	0.1		3.88	0.30	0.0000 ↑	0.0670
	1		4.57	0.07	0.0042 ↑	0.2451
DptA	control	10	7.53	0.28	n/a	n/a
	0.1		8.37	0.35	n/a	0.3543
	1		7.35	0.58	n/a	0.8335
	control	20	5.94	0.55	0.0938	n/a
	0.1		4.24	0.32	0.0000 ↑	0.0725
	1		5.07	0.15	0.0157 ↑	0.3388
CecA1	control	10	0.43	0.14	n/a	n/a
	0.1		0.10	0.17	n/a	0.7140
	1		0.77	0.04	n/a	0.6895
	control	20	1.11	0.21	0.4676	n/a
	0.1		0.30	0.20	0.8106	0.4004
	1		0.94	0.10	0.8395	0.8342
dpp	control	10	11.10	0.38	n/a	n/a
	0.1		14.44	1.55	n/a	0.0002 ↓
	1		11.77	1.25	n/a	0.4601
	control	20	12.68	0.80	0.0875	n/a
	0.1		11.44	0.30	0.0009 ↑	0.1663
	1		13.10	1.35	0.1355	0.6148

C (μM) – Concentration (μM); dCt – delta Ct (cycle thresholds) values were calculated as the differences in Ct values for target genes and reference genes (*β-Tubulin* and *RpL32*). The Ct values are inversely proportional to the mRNA transcript levels. Higher dCt values represent a lower expression level. SE – standard error of mean; DT (p) – p-values for the Duncan's post hoc test; AE – effect of age; TE – effect of treatment, ↑ – increase, ↓ – decrease; n/a – not applicable.
