# Supplementary material for: Studying the Geroprotective Properties of YAP/TAZ Signaling Inhibitors on Drosophila melanogaster Model
Source: Int J Mol Sci. 2023 Mar 22;24(6):6006. doi: 10.3390/ijms24066006 (PMC10058302; doi:10.3390/ijms24066006)
Supplement: Supplementary file 1 [file ijms-24-06006-s001.zip › Table S14.pdf]

Table S14. Effect of Yap/Taz inhibitors on the expression level of antimicrobial genes *AttA*, *DptA*, *CecA1* and Wnt-related gene *dpp* in *Drosophila* females.

Gene	C (μM)	Age (days)	Mean (dCt)	SE	DT (p) AE	DT (p) TE
Verteporfin						
Atta	control	10	8.07	0.12	n/a	n/a
	0.01		8.00	0.12	n/a	0.815
	0.1		8.31	0.07	n/a	0.452
	control	20	6.05	0.10	0.000 ↑	n/a
	0.01		5.68	0.18	0.000 ↑	0.272
	0.1		6.71	0.34	0.000 ↑	0.065
DptA	control	10	8.61	0.24	n/a	n/a
	0.01		8.55	0.16	n/a	0.845
	0.1		9.33	0.18	n/a	↑ 0.047
	control	20	4.65	0.25	0.000 ↑	n/a
	0.01		3.91	0.33	0.000 ↑	↑ 0.021
	0.1		5.71	0.29	0.000 ↑	↓ 0.001
CecA1	control	10	9.26	0.17	n/a	n/a
	0.01		9.23	0.06	n/a	0.909
	0.1		9.16	0.09	n/a	0.771
	control	20	6.37	0.13	0.000 ↑	n/a
	0.01		6.69	0.06	0.000 ↑	0.339
	0.1		6.69	0.21	0.000 ↑	0.309
dpp	control	10	8.38	0.07	n/a	n/a
	0.01		9.32	0.68	n/a	↓ 0.009
	0.1		7.99	0.10	n/a	0.284
	control	20	7.14	0.12	0.000 ↑	n/a
	0.01		6.90	0.20	0.000 ↑	0.469
	0.1		6.72	0.04	0.000 ↑	0.222
ML-7						
Atta	control	10	7.08	0.21	n/a	n/a
	0.1		6.22	0.07	n/a	↑ 0.00
	1		7.34	0.26	n/a	0.38
	control	20	7.01	0.07	0.798	n/a
	0.1		6.00	0.29	0.425	↑ 0.00
	1		5.66	0.12	0.000 ↑	↑ 0.00
DptA	control	10	5.26	0.22	n/a	n/a
	0.1		4.87	0.29	n/a	0.18
	1		5.95	0.24	n/a	↓ 0.02
	control	20	5.09	0.33	0.535	n/a
	0.1		3.54	0.16	0.000 ↑	↑ 0.00
	1		3.76	0.18	0.000 ↑	↑ 0.00
CecA1	control	10	7.58	0.35	n/a	n/a
	0.1		7.92	0.10	n/a	0.24
	1		8.44	0.24	n/a	↓ 0.00
	control	20	7.57	0.09	0.990	n/a
	0.1		6.33	0.11	0.000 ↑	↑ 0.00
	1		7.08	0.11	0.000 ↑	0.11
dpp	control	10	8.19	0.05	n/a	n/a
	0.1		8.11	0.09	n/a	0.75
	1		8.50	0.28	n/a	0.30
	control	20	7.88	0.13	0.311	n/a

	0.1		7.52	0.17	0.065	0.25
	1		7.56	0.09	0.002 ↑	0.28
Cytohalasin D						
Atta	control	10	8.96	0.38	n/a	n/a
	0.1		9.22	0.16	n/a	0.62
	1		10.32	0.18	n/a	↓ 0.02
	control	20	6.95	0.35	0.001 ↑	n/a
	0.1		7.88	0.16	0.033 ↑	0.14
	1		7.21	0.35	0.000 ↑	0.66
DptA	control	10	10.86	0.07	n/a	n/a
	0.1		8.41	0.12	n/a	↑ 0.00
	1		11.38	0.24	n/a	0.33
	control	20	4.52	0.05	0.000 ↑	n/a
	0.1		6.07	0.29	0.000 ↑	↑ 0.00
	1		5.38	0.50	0.000 ↑	0.11
CecA1	control	10	9.56	0.17	n/a	n/a
	0.1		8.52	0.21	n/a	0.08
	1		8.89	0.19	n/a	0.25
	control	20	7.11	0.61	0.000 ↑	n/a
	0.1		7.62	0.32	0.166	0.36
	1		8.12	0.31	0.219	0.10
dpp	control	10	8.29	0.31	n/a	n/a
	0.1		8.29	0.21	n/a	1.00
	1		7.87	0.15	n/a	0.49
	control	20	9.95	1.11	0.006 ↓	n/a
	0.1		8.23	0.12	0.916	↑ 0.00
	1		7.79	0.20	0.875	↑ 0.00
AICAR						
Atta	control	10	8.81	0.32	n/a	n/a
	0.1		9.65	0.14	n/a	0.11
	1		8.60	0.29	n/a	0.68
	control	20	6.48	0.08	0.000 ↑	n/a
	0.1		5.35	0.34	0.000 ↑	↑ 0.05
	1		5.51	0.39	0.000 ↑	0.08
DptA	control	10	9.31	0.45	n/a	n/a
	0.1		10.09	0.27	n/a	0.13
	1		6.29	0.64	n/a	↑ 0.00
	control	20	5.65	0.42	0.000 ↑	n/a
	0.1		5.70	0.46	0.000 ↑	0.92
	1		4.71	0.26	0.004 ↑	0.09
CecA1	control	10	8.27	0.23	n/a	n/a
	0.1		8.78	0.16	n/a	0.32
	1		8.17	0.42	n/a	0.85
	control	20	5.98	0.05	0.000 ↑	n/a
	0.1		4.71	0.35	0.000 ↑	↑ 0.02
	1		5.16	0.69	0.000 ↑	0.15
dpp	control	10	10.36	0.16	n/a	n/a
	0.1		10.97	0.42	n/a	0.24
	1		10.58	0.26	n/a	0.65
	control	20	12.08	0.32	0.001 ↓	n/a
	0.1		11.68	0.13	0.149	0.45
	1		11.74	0.32	0.029 ↓	0.49

C (μM) – Concentration (μM); dCt – delta Ct (cycle thresholds) values were calculated as the differences in Ct values for target genes and reference genes (*β-Tubulin* and *RpL32*). The Ct values are inversely proportional to the mRNA transcript levels. Higher dCt values represent a lower expression level. SE – standard error of mean; DT (p) – p-values for the Duncan's post hoc test; AE – effect of age; TE – effect of treatment, ↑ – increase, ↓ – decrease; n/a – not applicable.
