# Supplementary material for: Studying the Geroprotective Properties of YAP/TAZ Signaling Inhibitors on Drosophila melanogaster Model
Source: Int J Mol Sci. 2023 Mar 22;24(6):6006. doi: 10.3390/ijms24066006 (PMC10058302; doi:10.3390/ijms24066006)
Supplement: Supplementary file 1 [file ijms-24-06006-s001.zip › Table S15.pdf]

Table S15. List of primers.

Gene	Symbol (FlyBase)	Forward/Reverse (5'-3')
β-Tubulin at 56D	*β-Tubulin*	GCAACTCCACTGCCATCC/ CCTGCTCCTCCTCGAACT
Ribosomal protein L32	*RpL32*	GAAGCGCACCAAGCACTTCATC/ CGCCATTTGTGCGACAGCTTAG
Cyclin E	*CycE*	AAGCAGCGATTCAAACGAGGAC/ AGGAGGCAACCGATGACAGATTG
division abnormally delayed	*dally*	TCTACACGACCATCGTGGACAAC/ AGGGCACCTCGGGATTATATCG
Death-associated inhibitor of apoptosis 1	*Diap1*	AAGCCCTTCACCGATGTGATGC/ TTGAGGACTTGGGTGCGCATTG
E2F transcription factor 1	*E2f1*	TCAACGACATTGGCTCGGACTAC/ GCGGATTGATGGACACAAAGGC
Myc	*myc*	ACGGAAACTATGTTCAGCGAGAC/ TACGGATCAGAGCGGTAAAGGG
vein	*vn*	AGTGCCGGGCCAAGAATAAAGC/ TCCGTGGGAAAGTGAACTGGTG
wingless	*wg*	ACCCGATCAGCAAGATCCATCAC/ ATCTATTATGCTTGCGTCCCTGAC
yorkie	*Yki*	CCCAGAATCCAATATCGCCAGCAG/ AGCACGCTAAGCCCAGATTCATTC
decapentaplegic	*dpp*	GACGAAAGGGTATTCAAACT/ TTAGAACGCATAACGATGTAG
Attacin-A	*AttA*	TGCTCGTTTGGATCTGACCAAGG/ AAAGAGGCACCATGACCAGCATTG
Diptericin A	*DptA*	TTCACCATTGCCGTCGCCTTAC/ ATGTCGTCGGGCATCGGATAAG
cecropin A1	*CecA1*	TCGCTCAGACCTCACTGCAATATC/ TGTCCAATGGTGATGGCCAGAATG
